# Steroid Hormone Signaling Is Essential to Regulate Innate Immune Cells and Fight Bacterial Infection in *Drosophila*

**DOI:** 10.1371/journal.ppat.1003720

**Published:** 2013-10-24

**Authors:** Jennifer C. Regan, Ana S. Brandão, Alexandre B. Leitão, Ângela Raquel Mantas Dias, Élio Sucena, António Jacinto, Anna Zaidman-Rémy

**Affiliations:** 1 Instituto de Medicina Molecular, Faculdade de Medicina de Lisboa, Lisboa, Portugal; 2 Centro de Estudos de Doenças Crónicas, Faculdade de Ciências Médicas, FCM, Universidade Nova de Lisboa, Lisboa, Portugal; 3 Instituto Gulbenkian de Ciência, Oeiras, Portugal; 4 Universidade de Lisboa, Faculdade de Ciências, Departamento de Biologia Animal, Edifício C2, Lisboa, Portugal; Stanford University, United States of America

## Abstract

Coupling immunity and development is essential to ensure survival despite changing internal conditions in the organism. *Drosophila* metamorphosis represents a striking example of drastic and systemic physiological changes that need to be integrated with the innate immune system. However, nothing is known about the mechanisms that coordinate development and immune cell activity in the transition from larva to adult. Here, we reveal that regulation of macrophage-like cells (hemocytes) by the steroid hormone ecdysone is essential for an effective innate immune response over metamorphosis. Although it is generally accepted that steroid hormones impact immunity in mammals, their action on monocytes (e.g. macrophages and neutrophils) is still not well understood. Here in a simpler model system, we used an approach that allows *in vivo*, cell autonomous analysis of hormonal regulation of innate immune cells, by combining genetic manipulation with flow cytometry, high-resolution time-lapse imaging and tissue-specific transcriptomic analysis. We show that in response to ecdysone, hemocytes rapidly upregulate actin dynamics, motility and phagocytosis of apoptotic corpses, and acquire the ability to chemotax to damaged epithelia. Most importantly, individuals lacking ecdysone-activated hemocytes are defective in bacterial phagocytosis and are fatally susceptible to infection by bacteria ingested at larval stages, despite the normal systemic and local production of antimicrobial peptides. This decrease in survival is comparable to the one observed in pupae lacking immune cells altogether, indicating that ecdysone-regulation is essential for hemocyte immune functions and survival after infection. Microarray analysis of hemocytes revealed a large set of genes regulated at metamorphosis by EcR signaling, among which many are known to function in cell motility, cell shape or phagocytosis. This study demonstrates an important role for steroid hormone regulation of immunity *in vivo* in *Drosophila*, and paves the way for genetic dissection of the mechanisms at work behind steroid regulation of innate immune cells.

## Author Summary

Hormones orchestrate physiological processes such as metabolism, immunity and development, and allow the body to respond systemically to external stresses and resources. Steroid hormones, such as sex hormones and corticosteroids, affect the function of the immune system, but how they do so is not well understood. The fruit fly *Drosophila* relies on the innate immune system to protect itself from infection by microbes. The high level of functional conservation of *Drosophila* immune cells, or hemocytes, with mammalian monocytes, and the powerful genetic tools makes the fly a useful model to uncover new aspects of innate immunity. Here, we have analyzed the regulation of hemocytes by the steroid hormone ecdysone at the moment when the juvenile larval form begins the process of metamorphosis, the drastic change of body plan that gives rise to an adult fly. We reveal that ecdysone rapidly activates hemocytes, increasing their ability to move, respond to wounds and engulf dead cells or invading bacteria. Hemocytes rendered insensitive to the hormone are not activated and as a result, the fly becomes susceptible to bacterial infections, compromising its survival. This study contributes to our understanding of steroid regulation of monocyte function, which has broad implications for human immunity.

## Introduction

Immunity is essential for survival, yet energetically expensive and potentially self-damaging. The immune system needs to be tightly regulated and highly responsive to changes in external and internal environments, adapting to developmental stage [1], nutritional state [2], and stress [3]. Hormonal control of the immune system is not well-understood, and lies at the heart of a diverse range of clinically relevant phenomena including immune circadian rhythm [4], [5], obesity induced inflammation [6] and age- and gender-differences in immune function [1], [7].

*Drosophila* has proven to be a fruitful model of innate immunity [8]. The innate immune system in the fly comprises humoral and cellular responses [8]. The humoral response is best characterized by the secretion of antimicrobial peptides (AMPs) locally by epithelia such as the gut, or systemically by the fat-body, a functional analogue of the mammalian liver. This response to bacterial or fungal infection is mainly regulated by the Toll and Imd signaling pathways. Cellular immunity is performed by hemocytes, phagocytic circulating immune cells. *Drosophila* has become a powerful system to study phagocytosis due to high conservation of the processes involved [9], [10]. Cellular immunity is required to survive various types of bacterial infections, complementing the humoral response [11]–[20] and is involved in the response to wasp parasitoid infestation [21]. In addition, in the larvae, hemocytes play a role in inter-organ communication, as they are required for the fat-body to mount a full humoral antimicrobial response after an intestinal infection [12], [22], [23]. Hemocytes are also recruited to wounds in embryo and larvae [24]–[26], potentially clearing damaged tissue and preventing further dispersal of microorganisms from unsterile wound sites. In parallel to their role in immunity, hemocytes perform developmental and homeostatic functions such as the phagocytosis of apoptotic cells and extracellular matrix secretion. These functions are essential at embryonic stages, for example, for central nervous system and renal tubule development [27], [28]. The crosstalk between immunity and nutritional state or stress [2], [29]–[31] in *Drosophila* is beginning to be unravelled, however, developmental regulation of the immune system remains enigmatic.

Ecdysone is a steroid hormone, similar to mammalian estrogens and androgens; peaks in ecdysone titer regulate the major developmental transitions in the fly, including metamorphosis. The pupal stage lasts 4 days from the end of the 3^rd^ larval instar, after which adult flies eclose [32]. The biologically active form of ecdysone, 20-hydroxyecdysterone (20-E, hereafter referred to as ‘ecdysone’) coordinates tissue remodelling at metamorphosis [32]. This hormone activates a nuclear receptor, the Ecdysone Receptor (EcR), which acts as a heterodimer with its partner Ultraspiracle (USP), a homologue of the mammalian Retinoid X Receptor. Together, they activate the transcription of primary response genes, which in turn activate the transcription of a battery of late response genes [32]. This transcriptional cascade ultimately leads to the induction of both cell death in larval tissues and differentiation and proliferation of the imaginal discs into adult tissues [32]. In addition, several lines of evidence indicate that ecdysone regulates some aspects of hemocyte behaviour. In the larva, while the majority of hemocytes are in circulation, approximately one third of the total population interacts with tissues, attaching in repeated patches to the dorsal epithelium along the longitudinal axis [33]. Despite the fact that hemocytes from these patches are mostly immotile at larval stages, it has been noticed that they disperse at metamorphosis [33]. This observation correlates with the recent finding that *ex vivo*, hemocytes activate motility and morphological changes after metamorphosis [34]. Cell shape changes can be prematurely triggered in larvae by ecdysone injection [33]. Last, it is long known that ecdysone treatment is important to potentiate AMP gene expression and phagocytosis after an immune challenge in hemocyte-derived cell culture lines [35]. Although these results suggest an ecdysone-dependent regulation of hemocyte function, *in vivo* evidence of a direct effect of ecdysone signaling on hemocyte behaviour, and its functional relevance, is lacking.

Here, we explore the hormonal regulation of *Drosophila* hemocytes at metamorphosis and its impact on *Drosophila* immunity using an *in vivo* approach. We demonstrate that direct activation of ecdysone signaling in hemocytes is necessary to increase their developmental and immune activities at metamorphosis, including phagocytosis. We show that this activation is essential to respond efficiently to and survive pathogenic challenge.

## Results

### 1. The Ecdysone Receptor is required in the hemocytes for their activation at metamorphosis

**Figure 1:**
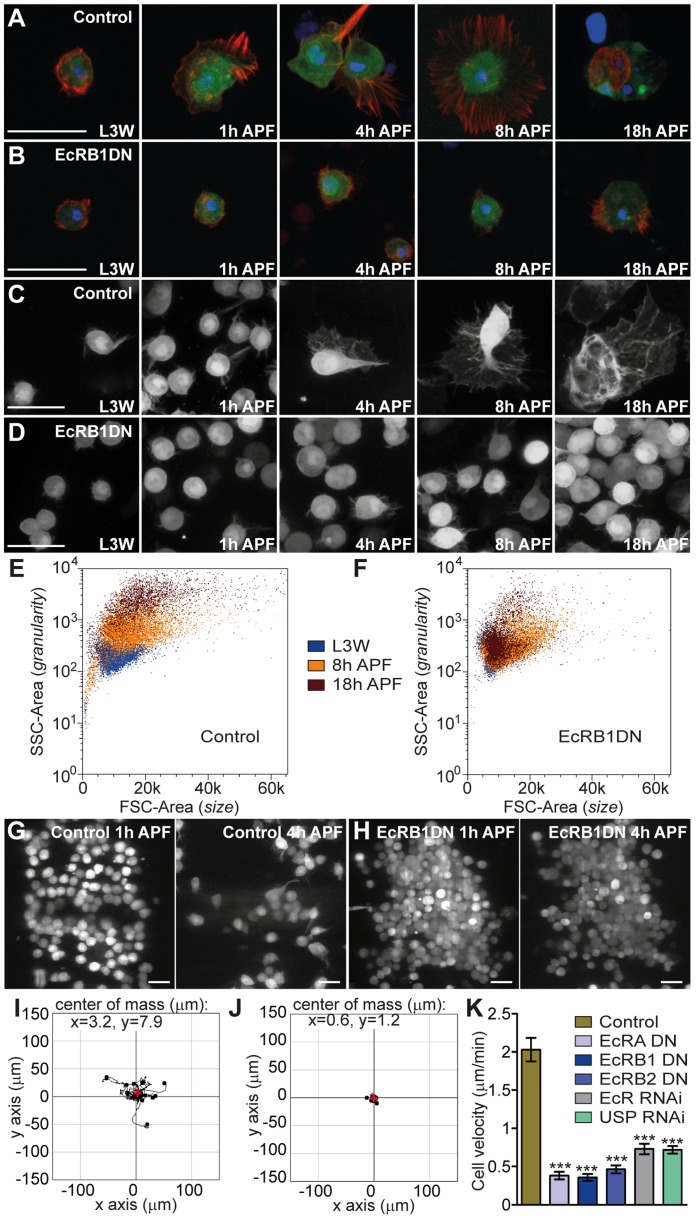
Ecdysone signaling is required for hemocyte activation at metamorphosis. (A–D) Analysis of the morphology of control hemocytes (A, C) and hemocytes expressing a DN form of the EcR receptor (EcRB1DN; B, D) at precise time points before and after puparium formation (APF). (A, B) *Ex vivo* analysis of bleeds; (C, D) *in vivo* analysis of cells visualized under the dorsal epithelium. Green, endogenous GFP. Blue, DAPI. Red, phalloidin. (E,F) Forward scatter (FSC)–Area/side scatter (SSC)–Area plots reflecting size (x axis) and granularity (y axis) of hemocytes retrieved from L3W, 8 h APF and 18 h APF control (E) and *HmlΔ>EcRB1DN* (F) animals. (G–K) Hemocytes insensitive to ecdysone do not activate motility nor disperse at metamorphosis (data retrieved from *in vivo* time-lapse imaging; see typical movies 1 and 2). (G,H) Epithelia-associated (‘sessile patch’) hemocytes are visible as groups of cells at 1 h APF, but have largely dispersed by 4 h APF in control animals (G). *EcRB1DN*-hemocytes do not undergo dispersal (H). (I,J) Tracks corresponding to the trajectories of twenty cells, for 80 min (starting 2h40 APF) were superimposed at (0;0). Center of mass of all endpoint positions is marked with a red cross (x/y coordinates indicated on figure) and indicates a random migration of control hemocytes (I; center of mass not significantly different from (0;0)). *EcRB1DN*-hemocytes are largely immotile (J). (K) Cell velocity measured at 1 h APF. Mean and SEM are displayed. Scale bars represent 20 µm in all panels.

To test whether ecdysone signaling cell-autonomously regulates hemocyte shape changes at metamorphosis, we used the *Hml-(Hemolectin-)ΔGal4* driver [36] to specifically express green fluorescent protein (GFP) and dominant-negative constructs of the three known EcR isoforms under the control of the UAS sequence. We imaged hemocytes *ex vivo* by bleeding larvae or pupae at precise time points after puparium formation (APF) (Fig. 1A–B) and *in vivo* through the dorsal epidermis (Fig. 1C–D). The first hour of the 12h ‘prepupal’ period is characterized by a translucent pupal case (which then darkens). Control hemocytes (from *HmlΔGal4, UAS-GFP/+* pupae, here after referred to as *HmlΔ*>GFP) displayed a clear change of morphology over metamorphosis: they became gradually more polarized, with many cytoplasmic protrusions and a higher number of vacuoles, increasing in size dramatically, likely due to their greater spread and many phagocytic vesicles (Fig. 1A and C). In striking contrast, hemocytes expressing a dominant negative (DN) form of the EcRB1 isoform (from *HmlΔGal4, UAS-GFP/UAS-EcRB1DN* pupae, here after referred to as *HmlΔ>EcRB1DN*) did not show any obvious change of size or morphology (Fig. 1B and D). Similar results were obtained when we analysed by flow cytometry the properties of the hemocyte population at the onset of pupariation. Control hemocytes displayed a clear shift both in Forward Scatter (FSC) and Side Scatter (SSC), indicating an increase in cell size and granularity, respectively (Fig. 1E and S1). In contrast, the FSC and SSC of hemocytes expressing *EcRB1DN* remained stable over metamorphosis and similar to the parameters observed for control hemocytes in late 3^rd^ instar larvae (L3 wandering; L3W; Fig. 1F and S1).

In control pupae, the cell shape changes were concomitant with a dispersal of the dorsal patches of hemocytes visualised by *in vivo* cinemicroscopy and cell tracking analysis (Fig. 1G–J). These so-called ‘sessile hemocytes’ changed their behaviour at pupariation and began to actively migrate under the epithelium (see video 1 and Fig. 1G, I). This dispersal did not occur when hemocytes expressed EcRB1DN (Fig. 1H, J and video 2). In these individuals, hemocytes were more numerous, which correlated with higher proliferation at larval stages (Fig. S2). Moreover, they were unable to migrate away from their original location (Fig. 1J). Measurements of cell velocity clearly indicated an impaired motility in EcRB1DN-expressing hemocytes, as compared to control hemocytes (Fig. 1K). Importantly, this defect in motility was not due to a decrease in viability, and appeared specific to the pupal stage as neither localisation of hemocytes to the gut in larvae, nor embryonic hemocyte migration were affected when EcRB1DN was expressed specifically in hemocytes ([26], [37]; Fig. S3). In addition, when we expressed EcRB1DN just before the initiation of puparium formation, as opposed to throughout larval stages, we also saw a significant reduction in motility (Fig. S4). The expression of dominant negative constructs for each of the three isoforms of EcR showed a similar phenotype to the EcRB1 isoform (Fig. 1K). EcR function in the regulation of hemocyte motility was further confirmed by RNAi (Fig. 1K). Last, the expression of *USP* RNAi in the hemocytes led to a similar lack of motility (Fig. 1K), indicating that the functional ecdysone receptor complex (EcR/USP) is required for this process.

### 2. Hemocytes insensitive to ecdysone are unable to perform dead cell phagocytosis during metamorphosis

**Figure 2:**
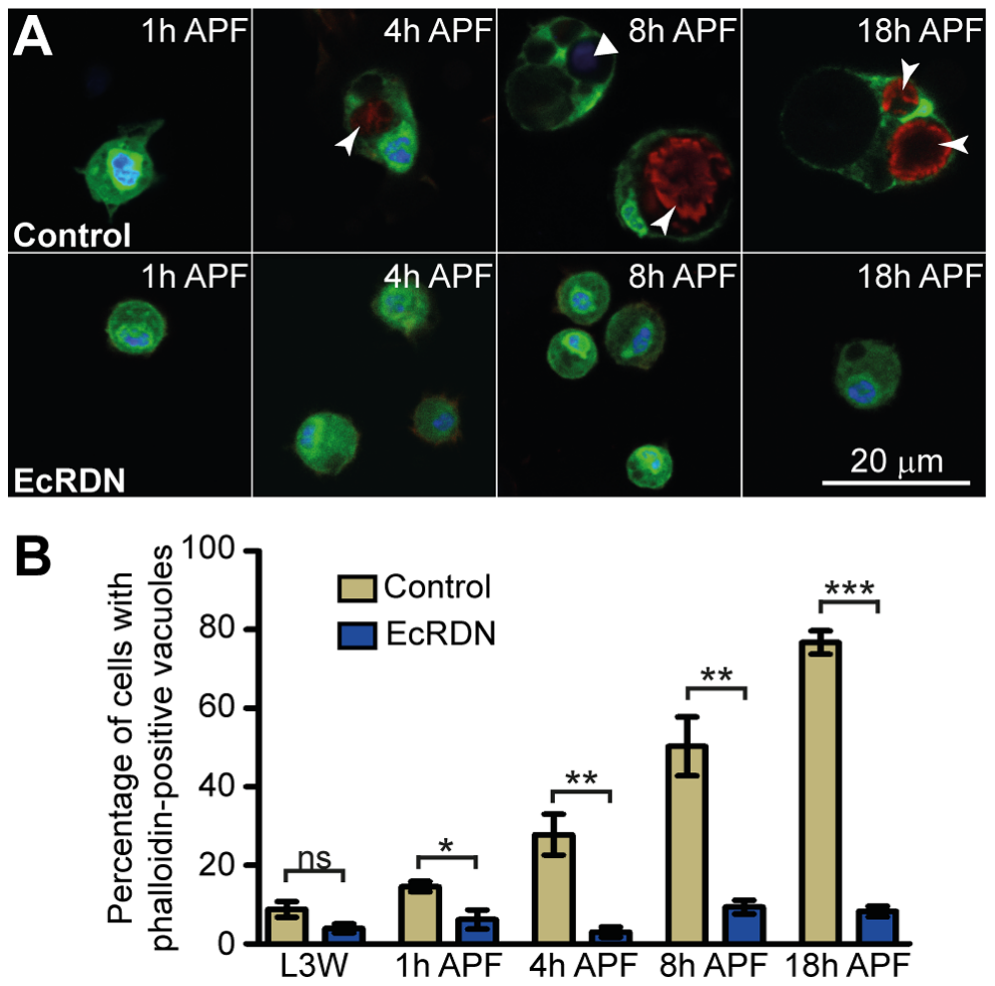
Ecdysone directly regulates hemocyte ability to phagocytose dead cells during metamorphosis. (A) *Ex vivo* analysis of circulating hemocytes at precise time points APF. Phagosomes containing dead cells (fragmented nuclei) and muscle fragments (striated phalloidin-positive inclusions) are indicated by solid and forked arrowheads, respectively. Green, endogenous GFP. Blue, DAPI. Red, phalloidin. Scale bars represent 20 µm. (B) Quantification of muscle cell engulfment. The graph displays the number of hemocytes with at least one phalloidin-positive vacuole (corresponding to muscle cell). For statistical analysis, significances based on t-test are indicated on the graph (comparison of control and EcRDN-expressing hemocytes at each developmental stage). One way-ANOVA was used to compare EcRDN-expressing hemocytes at different stages (ns) or control hemocytes at different stages (***). For control hemocytes, Tukey-Kramer post-test revealed significant differences between L3W and 8 h (***) or 18 h (***), between 1 h and 8 h (***) or 18 h (***), between 4 h and 8 h (*) or 18 h (***), and between 8 h and 18 h (**).

We next addressed the question of whether ecdysone signaling regulates ‘house keeping’ functions of hemocytes, required for homeostasis in the organism. During development, one major task for hemocytes is to scavenge and engulf dying cells [38]. We recovered hemocytes at different time points after the initiation of metamorphosis and stained them for DNA and F-actin. Hemocytes from control animals barely contained any stained vesicles at 1 h APF (Fig. 2A), but progressively presented a growing number of DNA and/or F-actin-containing vesicles (4 h, 8 h and 18 h APF; Fig. 2A). By 8 h APF, most of the control hemocytes had engulfed up to ten apoptotic cells, identifiable by the fragmented aspect of their nuclei, and/or huge pieces of muscles characterized by a strong, striated phalloidin staining. Such vesicles were not seen in the hemocytes expressing *EcRB1DN*, which were stained only for their own nucleus (Fig. 2A). We quantified the proportion of cells with phagosomes containing phalloidin-positive muscle fibres and found that the difference between control and *EcRB1DN* hemocytes was striking. In control pupae, we observed a significant increase in dead cell engulfment over time (p<0.001): just under 20% of hemocytes contained muscle tissue at 1 h APF, rising to approximately 80% of cells by 18 h APF (Fig. 2B). In *EcRB1DN* hemocytes the proportion was <10% at 1 h APF and remained at this low level throughout early pupal stages (Fig. 2B; no statistical differences between the five stages). The difference between *EcRB1DN* and control hemocytes was significant at every stage (Fig. 2B). Despite this effect on phagocytosis of larval tissue, viability of *HmlΔ>EcRB1DN* individuals from prepupal stage to adulthood was not affected as compared to control individuals, nor was their duration of metamorphosis (Fig. S5).

### 3. Activation of hemocytes at metamorphosis is important for survival after infection

**Figure 3:**
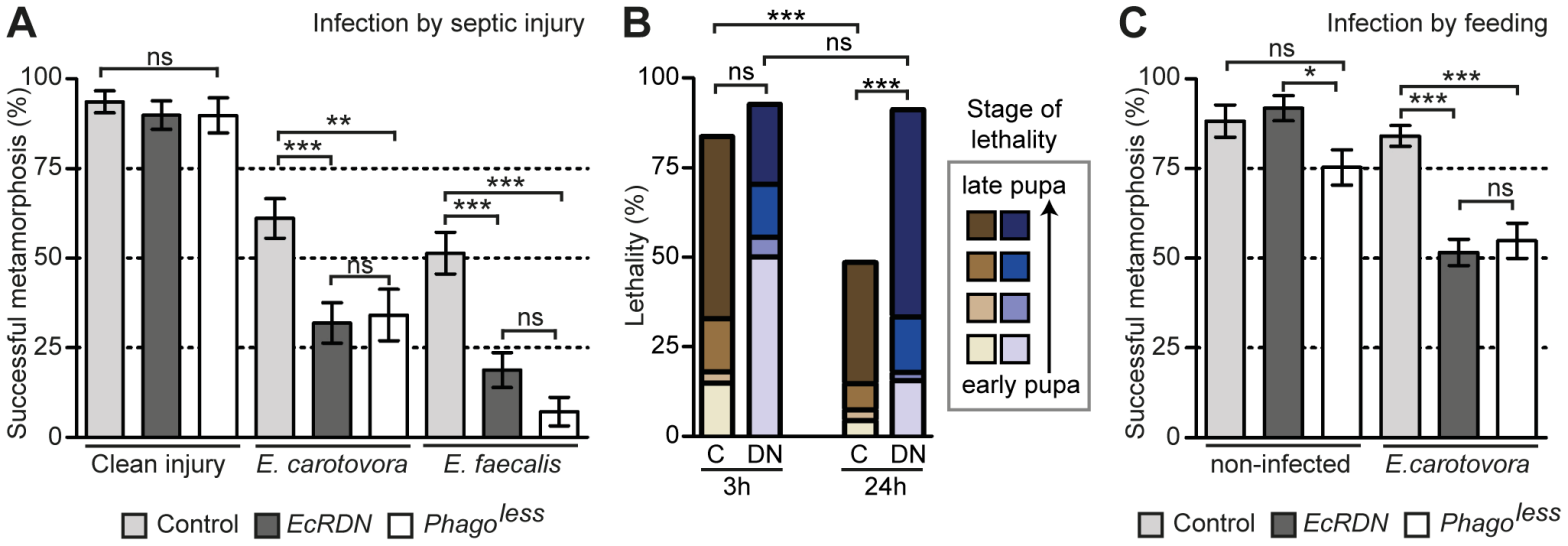
Pupae with ecdysone-insensitive hemocytes are susceptible to septic injury and oral infection. (A) Survival to septic injury with Gram-negative (*E. carotovora*) and Gram-positive (*E. faecalis*) bacteria. (B) Stage of lethality after septic injury with *E. carotovora*. Control or *HmlΔ>GFP/EcRB1DN* pupae were carefully staged and infected by septic injury with *E. carotovora* (O.D. 100) at 3 h or 24 h APF. Proportions of individuals dying at different stages over metamorphosis were determined by ‘post-mortem’ examination of the pupae. Pupae were classified into four arbitral categories, based on their appearance at arrested development: “early”, “intermediate 1”, “intermediate 2”, and “late” stage, here represented by a colour gradient (the darker the color, the older the pupa at time of death). The “late” stage corresponds to pupae that look ready to eclose, but finally did not emerge. Statistical analysis displayed on the graph corresponds to Wilcoxon test made without taking into account the stage of death but only the final survival over metamorphosis. An additional analysis with Wilcoxon test taking into account the stage of death indicates significant differences between control and EcRDN at both time point (***), between control at 2 h and 24 h (***) and between EcRDN at 2 h and 24 h (***). (C) Survival to oral infection performed at larval stage with *E. carotovora* bacteria. Control: *HmlΔ>GFP/+* pupae; EcRB1DN: *HmlΔ>GFP/EcRB1DN* pupae; ‘*Phago^less^*’: *HmlΔ>GFP/UAS-Bax*. Graphs display mean and SEM.

To find out if hemocyte activation by ecdysone is important in critical situations, such as wounding or infection, we tested the ability of *HmlΔ>EcRB1DN* pupae to resist clean or septic injuries with the Gram-negative bacteria *Erwinia carotovora* (*E.carotovora*) or the Gram-positive bacteria *Enteroccocus faecalis* (*E.faecalis*) when wounded at end of prepupal/early pupal stage (10 h–30 h APF). Interestingly, *HmlΔ>EcRB1DN* pupae survived as well as control individuals to clean injury, but were significantly less resistant to both types of bacterial infections (Fig. 3A). In order to understand better if the difference in resistance between control and *HmlΔ>EcRB1DN* animals is more pronounced at later stages when hemocytes are fully activated in control individuals, we imposed Gram-negative septic infection on closely-staged pupae at either 3 h APF or 24 h APF. We found that the difference between overall survival of control and *HmlΔ>EcRB1DN* pupae wounded at 3 h APF was not significant, whereas *HmlΔ>EcRB1DN* pupae wounded at 24 h APF survived significantly less than controls (p<0.001; Fig. 3B). When we analysed the time of death of infected pupae more precisely, we saw that although the overall survival of *HmlΔ>EcRB1DN* and control pupae wounded at 3 h APF were not significantly different, *HmlΔ>EcRB1DN* pupae tended to succumb to infection much sooner than controls (Fig. 3B). Altogether, the data suggest that the difference in resistance between *HmlΔ>EcRB1DN* and control is more pronounced at later stages when control hemocytes are fully activated, although we cannot exclude the possibility that other differences between the two stages could have an effect on the severity of the infection.

We next tested whether feeding larvae with bacteria (‘natural infection’) would affect their survival over subsequent metamorphosis. Control individuals were not affected by the oral infection with *E. carotovora*, and eclosed normally (Fig. 3C). In contrast, we observed a significant decrease in the survival of *HmlΔ>EcRB1DN* pupae (Fig. 3C). For both septic and natural infections, the observed decreased in survival was similar to that caused by genetic ablation of hemocytes (by expression of the pro-apoptotic gene Bax in hemocytes [13]; see “Phago^less^” survival in Fig. 3A and C). Thus hemocyte activation at metamorphosis is essential for the efficient participation of the cellular immune response in resistance to bacterial infections.

### 4. Hemocytes require ecdysone signaling to become wound-responsive at metamorphosis

Because pupae in which hemocytes are not activated succumb to infections more often than controls, we surmise that ecdysone signaling has a role in the regulation of the hemocyte response to infections. Therefore, we analysed the three main known responses of hemocytes to infections to determine whether they were affected by ecdysone signaling: i) wound response, ii) participation in AMP systemic expression (inter-organ signaling) and iii) phagocytosis.

**Figure 4:**
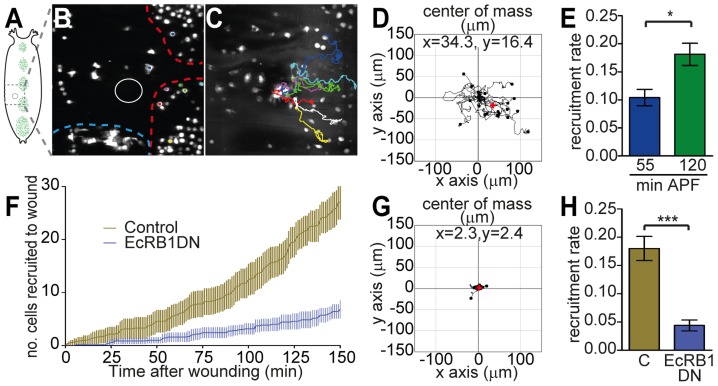
Acquisition of wound responsiveness at metamorphosis is dependent on ecdysone signaling. (A–E) Laser wounds were made in the epithelium of white prepupae (A, B). Red and blue dashed lines in (B) delineate central patches and lateral stripes of attached hemocytes, respectively. 200 min after wounding numerous hemocytes are visible at the wound (C); example tracks are shown (see video S3). Tracks of individual cells measured over 80 min, 70 min after wounding (wound performed at 1h30 APF) are superimposed at (0;0) in the tracking plot; all trajectories were rotated to maintain their relative positions towards the wound center (D). The center of mass of all tracks, indicated with red cross, is shifted towards the wound (positive *x*-axis) and significantly different from (0;0) (p = 0.01). Recruitment rate (cell/min) is significantly higher in pupae wounded at 120 min APF than in pupae wounded 55 min APF (E). Profile of hemocyte recruitment to epithelial wounds in control (brown) or *HmlΔ>EcRB1DN* pupae (blue) (F). Tracks corresponding to trajectories of *EcRB1DN*-hemocytes after epithelial wounding were superimposed and rotated as above in tracking plot (G); note that the center of mass (red cross) remains close to (0;0). Rate of recruitment (cell/min) in *HmlΔ>EcRB1DN* pupae and controls wounded 1h30 APF (H). Mean and SEM are displayed on graphs.

Upon injury, hemocytes are recruited to damaged tissues and wounded epithelia. This may be important to limit infection in the case of septic injury: once recruited to the wound, hemocytes phagocytose bacteria and prevent their spreading inside the organism. In the embryo, hemocyte recruitment involves active migration toward the wound by chemotaxis [25], [26]. In the larva by contrast, hemocytes are described as being recruited passively from the circulation by ‘capture’ at the wound site [24]. Hemocytes attached under the epithelium do not respond to wounds at larval stages, even to those in their close vicinity [24]. Interestingly, when we wounded the dorsal epithelium of prepupae using a laser-ablation system (which allows immediate high resolution imaging after wounding and tracking of hemocyte movement), we observed that most recruited cells had not come from circulation but by active migration under the epithelium, often from the so-called ‘sessile patches’ (Fig. 4A–C and video 3). Analysis of hemocyte tracks after wounding confirmed their directed migration towards the wound (Fig. 4D; compare with the center of mass close to (0;0) in the absence of a wound, indicative of a random migration, Fig. 1I). Importantly, the capacity of hemocytes to respond to wounds clearly increased over early prepupal stages. Indeed, hemocyte recruitment rate increased with pupal age: after wounding prepupae at 120 min APF, we observed a higher rate of recruitment of hemocytes to wounds as compared with prepupae wounded at 55 min APF (Fig. 4E). Strikingly, *EcRB1DN*-hemocytes remained unresponsive to wounds and very few were recruited to the sites of damage (Fig. 4F–H and video 4).

Thus, our data reveal that the mode of recruitment of hemocytes to wounds is modified at pupal stage, switching from a passive ‘capture’ of circulating cells in larvae [24], to active migration of hemocytes toward the wounds in pupae. This activation is lost in hemocytes insensitive to ecdysone.

### 5. Ecdysone signaling is required for bacterial phagocytosis and to fight infection at metamorphosis

Hemocytes are involved in the systemic induction of the Imd pathway after oral infection in the larva [12], [22], [23], [39], and therefore we next tested the hypothesis of an ecdysone-dependent regulation of “gut-to-fat-body” communication by hemocytes. However, when we analysed the expression of four AMPs (*Diptericin*, *Attacin*, *Metchnikowin* and *Drosomycin*) in prepupae after oral infection with *E. carotovora* at larval stage (Fig. S6), we found that *HmlΔ>EcRB1DN* prepupae induce a systemic humoral response similar to that of control. Thus, our data indicate that ecdysone signaling in hemocytes is not required for inter-organ communication after oral infection. In addition, we analyzed the local epithelial response to oral infection by examining expression of *Diptericin* specifically in the gut, and found that this response is also unaffected in *HmlΔ>EcRB1DN* prepupae (Fig. S7). We wondered if phagocytic activity, on the other hand, would be ecdysone-regulated at metamorphosis and could account for the observed decrease in survival to infection.

**Figure 5:**
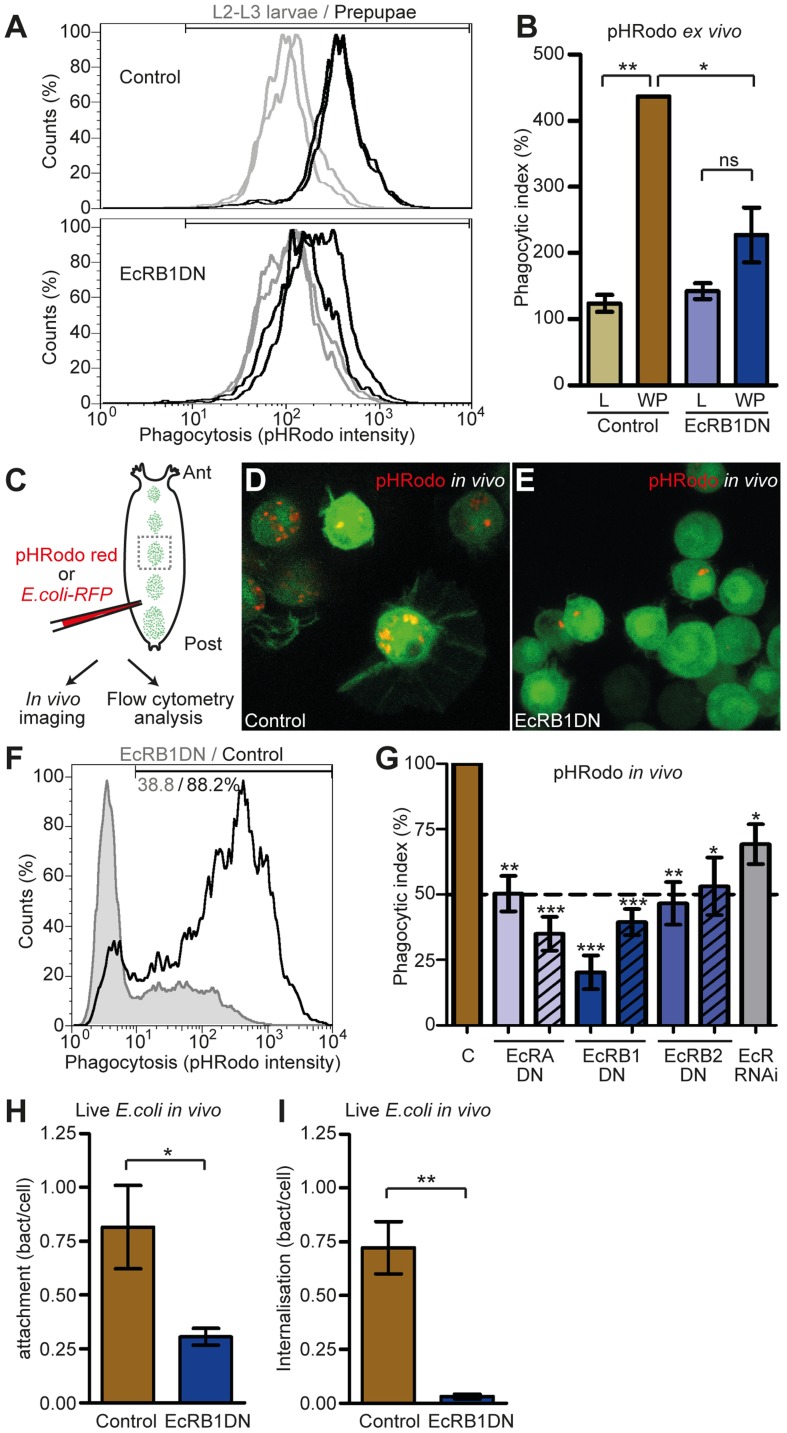
Bacterial phagocytosis is activated by ecdysone signaling at metamorphosis and is required to survive infection. (A, B) Ecdysone-dependent increase of phagocytic index at metamorphosis. Flow cytometry measurements of *ex vivo* phagocytosis (pHRodo fluorescence intensity) in hemocytes retrieved from larvae and white prepupae (A); top panel: control hemocytes; bottom panel: *EcRB1DN*-hemocytes. Phagocytic index (percentage of positive cells x mean intensity) of larval and prepupal control hemocytes or hemocytes expressing *EcRB1DN* (B). (C–I) Ecdysone signaling is required *in vivo* for full activation of phagocytosis at metamorphosis. Schematic demonstrating *in vivo* technique in white prepupa (C). Live imaging of pHRodo particles in control hemocytes (D), or *EcRB1DN*-expressing hemocytes (E). Flow cytometry measure of phagocytosis (F): pHRodo-fluorescence intensity profile of pHRodo-positive *EcRDNB1*-hemocytes (corresponding to 38.8% of total *EcRDNB1* hemocytes) and pHRodo-positive control hemocytes (88.2% of total control hemocytes). Phagocytic index (flow cytometry experiment) of hemocytes expressing DN forms of all three EcR isoforms or *EcR RNAi* (G). Striped and full-colour bars correspond to DN forms ^W650A^ and ^F645A^, respectively. *EcRDNB1*-hemocytes are also impaired in attachment (H) and internalization (I) indexes of injected live *E.coli-RFP* (*in vivo* live imaging experiments). Mean and SEM are displayed on graphs.

To first address this question, we bled late L2/young L3 larvae or young prepupae, incubated the retrieved hemocytes with a high concentration of pHRodo bacterial particles (that emit a strong fluorescent signal only at low pH, i.e. in the acidic environment of the phagosome) and assessed the ability of hemocytes to perform phagocytosis using flow cytometry (Fig. 5A–B). Strikingly, hemocytes presented a clear shift in their ability to perform phagocytosis from L2/L3 to prepupal stages (Fig. 5A); the prepupal hemocyte phagocytic index (number of phagocytosing hemocytes multiplied by mean pHRodo intensity per hemocyte)/total number of hemocytes) was almost four times the index of L2/L3 larval hemocytes (Fig. 5B). Moreover, *EcRB1DN*-hemocytes did not present such a shift and maintained the same phagocytic ability at prepupal stage as at L2/L3 larval stages (Fig. 5A–B).

To confirm this phenotype *in vivo*, we injected pHRodo bacterial particles in control and *HmlΔGal4>EcRB1DN* prepupae and imaged hemocytes live through the dorsal epithelium (Fig. 5C). While under these conditions, all hemocytes in control prepupae had performed phagocytosis of numerous particles, only very few *EcRB1DN*-hemocytes contained particles and then in low numbers (Fig. 5D–E). We complemented this analysis with a flow cytometry method that combines *in vivo* assay with high-throughput quantitative analysis. One hour after injection with pHRodo, hemocytes were retrieved from prepupae and analysed for their fluorescence by flow cytometry. In accordance with the results obtained with confocal analysis, fewer *EcRB1DN*-hemocytes were pHRodo-positive as compared to control (40% versus 88%; Fig. 5F). Those few hemocytes positive for pHRodo had a lower fluorescence intensity than control hemocytes (Fig. 5F), contributing to a global decrease in phagocytic index (Fig. 5G). Interestingly, hemocytes expressing dominant negative forms of any of the three EcR isoforms were affected in their ability to perform phagocytosis, as well as hemocytes expressing *EcR RNAi* (Fig. 5G).

As pHRodo particles only fully emit fluorescence once in the mature phagosome, the above results could also be indicative of a defect of *EcRDN*-hemocytes in phagosome maturation. To test this hypothesis we analysed the ability of control and *EcRB1DN*-hemocytes to bind and engulf live *Escherichia coli* (*E.coli*). *E.coli* expressing the red fluorescent protein (*E.coli-RFP*) were injected in prepupae and phagocytosis was monitored *in vivo* (video 5 and 6). *EcRB1DN*-hemocytes were significantly impaired in their ability both to bind (attachment index: the average number of bacteria bound per hemocyte; Fig. 5H) and engulf bacteria (internalisation index: the average number of bacteria ingested per hemocyte; Fig. 5I and S7). Altogether, our results demonstrate that EcR-dependent activation of hemocytes is required for optimal phagocytic activity of hemocytes at metamorphosis, and modulate their ability to both bind and internalise bacteria.

### 6. EcR transcriptionally regulates genes involved in phagocytosis

Our phenotypic analysis has revealed the importance of ecdysone signaling in the regulation of several hemocyte functions, including bacteria phagocytosis. To better understand the molecular basis of the ecdysone-dependent regulation of hemocyte biology, we performed a hemocyte-specific transcriptomic analysis, combining FACS and Affymetrix microarrays. We first compared the transcriptome of “control” hemocytes retrieved from *w; Hml>GFP/+* 3^rd^ instar larvae and prepupae. We selected late feeding 3^rd^ instar larvae prior to the peak of ecdysone that induces metamorphosis, and compared them with hemocytes retrieved from 1 h–2 h APF prepupae, the stage we have used for the majority of our phenotypic analyses. This allowed us to determine which genes are induced or repressed at metamorphosis in hemocytes. Comparison with *EcRDN* hemocytes allowed us to determine which of these genes are dependent on EcR signaling.

**Figure 6:**
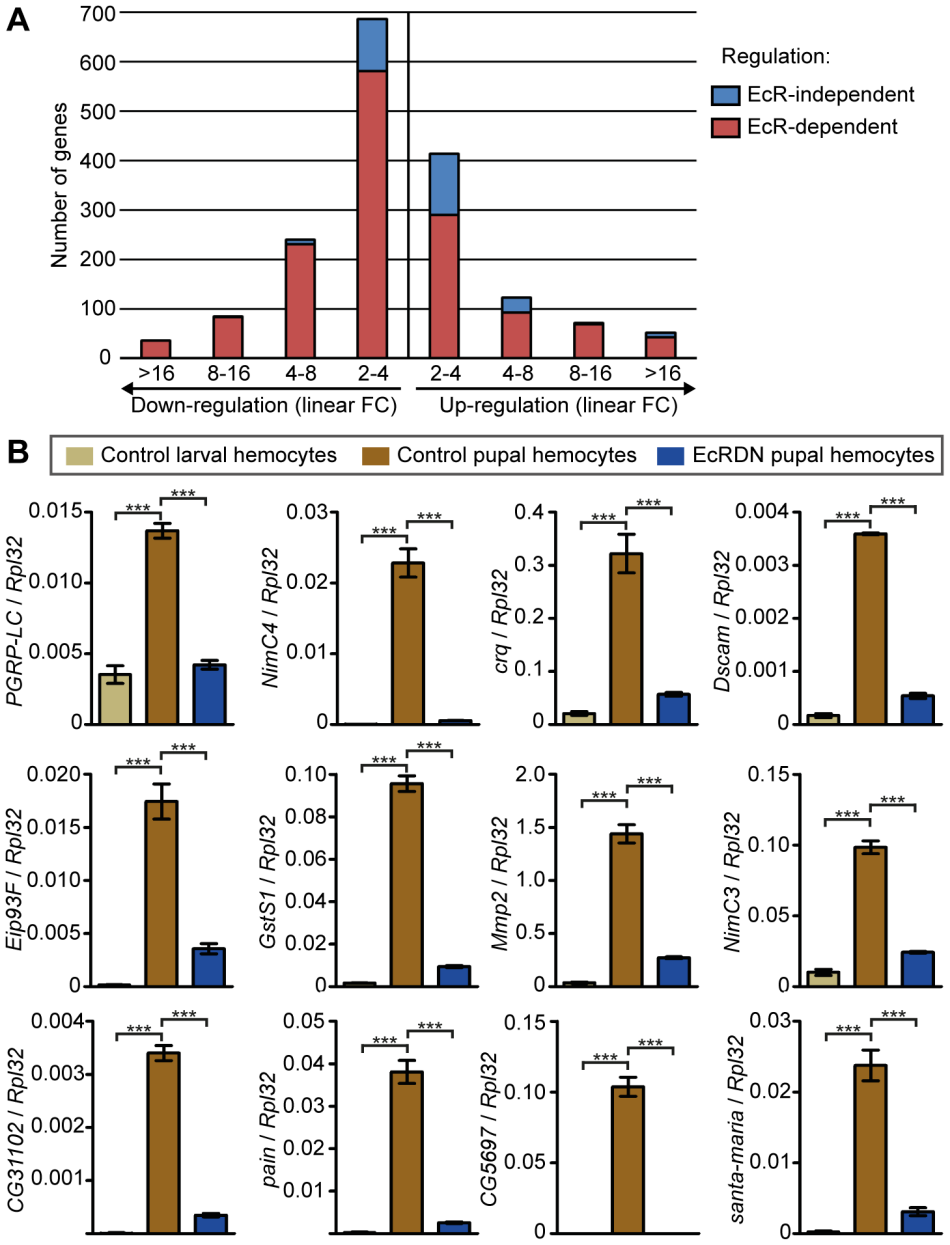
Transcriptomic analysis of the impact of ecdysone signaling on hemocytes at metamorphosis. (A) Distribution of the hit genes per fold change and per dependence to EcR. Genes whose expression is significantly (p<0.001) changed in hemocytes between late feeding larval 3^rd^ instar and early prepupae (1 h–2 h APF) were identified by microarrays and classified based on FC. FC Indicated are linear and only genes which FC>2 are represented on this figure. Up- or down-regulation of a gene was defined to be EcR-dependent when its expression was significantly altered in EcRB1DN-expressing pupal hemocytes (p<0.001; see Table S1 for gene list). (B) qPCR confirmation of the expression of 12 genes from the microarrays. qPCR was performed on RNA extracted from FACS-sorted hemocytes. The results displayed represent the mean and SEM of three biological repeats; samples were independent from samples used for microarrays. Statistical analysis was performed using one-way ANOVA and Tukey-Kramer post-test. For comparison between control larval hemocytes and control pupal hemocytes, as well as between control pupal hemocytes and EcRDN pupal hemocytes, significance are indicated on the graph. There was no significant difference between control larval hemocytes and EcRDN pupal hemocytes except for GstS1 (*), NimC3 (*), Mmp2 (*) and Dscam (***).

A high number of genes are transcriptionally regulated in hemocytes at the onset of metamorphosis (3331 genes, among which 659 are up-regulated more than two fold; p<0.001; see repartition by fold change in Fig. 6A. The full microarray data can be found in Table S2 and on GEO database accession GSE49326, http://www.ncbi.nlm.nih.gov/geo/query/acc.cgi?acc=GSE49326). Strikingly, our data indicate that most of the changes in gene expression are EcR-dependent at this stage (1955 genes out of 3331 are EcR-regulated; p<0.001; see repartition by fold change in Fig. 6A and the list of genes in Table S1). In fact, 83% of genes that present a fold change in expression greater than 2 at metamorphosis are regulated by EcR. The microarray analysis was further confirmed by hemocyte-specific quantitative PCR of 12 genes, whose expression was clearly up-regulated in control hemocytes at metamorphosis, and significantly lower in *EcRDN*-expressing hemocytes at the same stage (Fig. 6B). These data confirm and explain the huge impact of ecdysone signaling on hemocyte biology at the onset of metamorphosis. Interestingly, we discover that in prepupal hemocytes, ecdysone signaling up-regulates the expression of numerous genes involved in bacteria phagocytosis. Indeed, we found that 35 genes known to be involved in phagocytosis are significantly up-regulated in hemocytes from control pupae and significantly less expressed in hemocytes expressing *EcRDN* (Table 1). Among them, several genes encode phagocytic bacterial receptors (*Nimrod*, *Dscam*, *PGRP*-*LC*), a receptor for dead cell phagocytosis (*crq*), or are involved in the actin remodelling required for this process (such as *Rac2* and *SCAR*). As loss of expression of several of these genes has been shown to have a dramatic effect on phagocytosis, these molecular data are sufficient to explain how insensitivity to ecdysone in hemocytes results in the strong phagocytosis phenotype we describe. Similarly, we found several genes involved in cell motility or cell shape regulation to be up-regulated in the control hemocytes at metamorphosis, and not in *EcRDN*-expressing hemocytes (Table S3 and S4, respectively). These genes represent good candidates responsible for the phenotypes observed in our analyses; additionally, it is likely that this data contains further genes involved in phagocytosis, cell motility or cell shape as yet unrevealed.

**Table 1:** EcR transcriptionally regulates genes involved in phagocytosis.

Gene Symbol	Gene Accession	Control pupae vs control larvae		Control pupae vs EcRDN pupae		References on phagocytosis phenotype
		FC	p value	FC	p value	
NimC2	FBgn0028939	57.08	0	−4.40	0	[58] (Prediction)
**Eip93F**	FBgn0264490	26.40	0	−2.85	8.00E-06	[32] (Prediction)
scb	FBgn0003328	25.72	0	−1.98	1.30E-05	[57], [59]
Eip78C	FBgn0004865	11.88	0	−3.80	0	[9]
Tep2	FBgn0041182	9.19	0	−2.14	8.00E-06	[57]
**NimC4**	FBgn0260011	6.26	0	−5.34	0	[58] (Prediction)
**NimC3**	FBgn0001967	5.83	6.90E-05	−2.54	2.20E-05	[58] (Prediction)
**Mmp2**	FBgn0033438	5.81	8.00E-06	−1.68	5.50E-05	[9]
**Dscam**	FBgn0033159	3.96	1.10E-05	−2.75	1.80E-05	[60]
l(1)G0232	FBgn0028341	3.10	2.00E-06	−1.58	0.000193	[57]
yip2	FBgn0040064	3.01	7.00E-05	−1.70	0.000388	[9]
CG8449	FBgn0038129	2.74	5.00E-06	−1.59	0.000972	[9]
CG3638	FBgn0261444	2.67	2.00E-06	−1.74	2.40E-05	[57]
**crq**	FBgn0015924	2.60	5.30E-05	−1.75	4.40E-05	[61], [62]
mv	FBgn0265988	2.51	0.00043	−2.55	3.00E-06	[63]
CG16791	FBgn0038881	2.39	3.00E-06	−1.60	0.000161	[57]
flr	FBgn0260049	2.27	5.00E-06	−1.44	0.000183	[57]
cpb	FBgn0011570	2.25	5.00E-06	−1.48	0.00065	[55]
RhoGAP71E	FBgn0036518	2.19	8.00E-06	−1.48	0.000137	[56]
Rac2	FBgn0014011	2.03	9.00E-06	−1.43	0.000417	[9], [34], [55], [57], [64]
Arpc5	FBgn0031437	1.93	6.80E-05	−1.44	0.000494	[55], [57]
Snx3	FBgn0038065	1.93	1.80E-05	−1.35	0.00068	[57]
CG13887	FBgn0035165	1.92	1.90E-05	−1.41	0.000501	[9]
RN-tre	FBgn0020620	1.87	3.30E-05	−1.69	0.000108	[9], [56]
not	FBgn0013717	1.84	6.20E-05	−1.41	0.000369	[9]
**PGRP-LC**	FBgn0035976	1.81	6.50E-05	−1.75	0.000359	[56]
Mekk1	FBgn0024329	1.81	4.10E-05	−1.46	0.000132	[9]
kuz	FBgn0259984	1.79	4.80E-05	−1.36	0.000536	[57]
SCAR	FBgn0041781	1.63	1.00E-04	−1.34	0.00084	[57]
Vps35	FBgn0034708	1.57	0.00015	−1.37	0.000462	[9]
Traf4	FBgn0026319	1.57	0.00059	−2.26	3.00E-06	[57]
shark	FBgn0015295	1.53	0.00012	−1.36	0.00073	[65], [66]
mib2	FBgn0086442	1.53	0.00017	−1.45	0.000534	[9]
put	FBgn0003169	1.51	0.00013	−1.39	0.000902	[9]

Among the genes significantly up-regulated in control hemocytes at metamorphosis (p<0.001; linear FC and *p* values indicated on the table) and significantly down-regulated in EcRB1DN-expressing hemocytes (p<0.001; linear FC and *p* values indicated on the table), 35 genes were annotated by Flybase with the GO term phagocytosis or found in S2 cell-based RNAi screens for phagocytosis phenotype [9], [20], [55]–[57].

FC: linear Fold Change; in bold: genes for which expression pattern was confirmed by qPCR. All p values are <0.001, no FC threshold was applied.

## Discussion

Here we have demonstrated the cell-autonomous regulation of *Drosophila* phagocytes by a hormonal signal at metamorphosis. Our study provides *in vivo* evidence that the steroid hormone ecdysone regulates both developmental and immune functions of hemocytes, such as phagocytosis of dying cells and bacteria, and the acquisition of wound responsiveness at metamorphosis. Most importantly, non-activated hemocytes are unable to efficiently fight infections, reflecting the functional importance of this hormonal regulation for the organism.

Using an *in vivo* genetic approach to block EcR signaling specifically in hemocytes, we have shown that ecdysone directly regulates their cell shape. Moreover, our data indicates that ecdysone regulates the onset of hemocyte motility and dispersal at metamorphosis, reflecting its function in border cell motility during oogenesis [40]. Our microarray data reveal that EcR up-regulates the expression of several genes functioning in cell motility or cell shape regulation [41], [42], which could account for these phenotypes (Table S3 and S4). Arguably, migration of hemocytes between tissues is required for clearing dying larval tissues during the pupal period. We show that hemocytes expressing the *EcRDN* construct do not engulf dead cells, which is potentially a consequence of impaired phagocytosis, motility, or a combination of both, although we cannot distinguish between these possibilities. Ecdysone has previously been shown to induce the expression in the hemocyte-derived *mbn2* cell line of *croquemort* (*crq*; [35]), a gene encoding a receptor for apoptotic cells in the embryo [38]. *crq* was identified in our microarray analysis as showing EcR-dependent up-regulation at metamorphosis, and this was confirmed by qPCR, where *crq* expression is almost completely suppressed in *EcRDN*-expressing pupal hemocytes (Fig. 6B). The impaired expression of *crq* in *EcRDN* hemocytes likely contributes to their deficiency in apoptotic cell phagocytosis. Functionally, the regulation of hemocytes by ecdysone, which is the coordinator of larval tissue apoptosis, may be a smart way for the fly to synchronize its macrophage scavenging activity with the moment it is most needed, at metamorphosis. Surprisingly, we did not observe any gross developmental consequences of the loss of this function, whereby *HmlΔGal4>EcRB1DN* individuals completed metamorphosis without delay. This is in agreement with studies showing that under sterile conditions, pupae lacking hemocytes altogether progress normally through metamorphosis [12], [13]. It suggests that dead cells might be engulfed by other, non-professional phagocytes (e.g. neighbor cells as reported for tumorigenesis), cleared up by other unidentified means, or simply tolerated, in the absence of functional hemocytes.

Furthermore, we show that the activation of hemocyte motility at metamorphosis also correlates with a change in their response to induced epithelial damage. While in the larva hemocytes are passively recruited to wounds from circulation [24], we demonstrate that in the pupa they actively migrate to damaged tissues. Induction of epithelial wounds at different times APF demonstrated that active wound responsiveness is progressively acquired at metamorphosis. In agreement with previous *ex vivo* analysis [34], our data highlights an intriguing plasticity of hemocytes to adapt their migratory activity and their response to wounds throughout development: chemotaxis in embryos and pupae ([26] and this study) versus passive circulation and ‘capture’ to wounds in larvae [24]. This correlates with the observation that, although the heart is beating in a 20 h APF-old pupa, hemocytes are not propelled in the hemolymph by the heartbeat, but maintain a slow, steady, active migration on tissues (video 7).

Most importantly, our study provides the first *in vivo* evidence of hormonal regulation of the *Drosophila* cellular response to bacterial challenge. With both *ex vivo* and *in vivo* data, we have demonstrated an important role for EcR in the up-regulation of hemocyte phagocytic activity at metamorphosis (Fig. 5). How does ecdysone signaling regulate phagocytosis? Previous studies in hemocyte-derived cell lines have shown that ecdysone treatment increases the transcription of some immune-related genes encoding AMPs and immune receptors such as Crq [35], [43], [44]. Here, using a tissue-specific, whole genome transcriptomic approach, we demonstrate that many genes are regulated by ecdysone signaling in hemocytes at metamorphosis. This analysis reveals the molecular regulation behind the observed phenotypes and allows for the identification of candidate effector genes. For example, 35 genes up-regulated by EcR at metamorphosis have been previously attributed a function in phagocytosis. These genes encode proteins involved in different steps of the phagocytosis process, such as recognition (e.g. the receptors *PGRP-LC*, *croquemort*, and *Nimrod* family members, *Dscam* and *scab*), or cytoskeletal rearrangements required for the engulfment step (e.g. *RhoGAP71E*, *Rac2*, *Arpc5* and *SCAR*). Interestingly, PGRP-LC (FC 1.8 by microarray, 3.9 by qPCR) was recently shown to be induced in ecdysone-treated S2 cells [45]. It appears that ecdysone can regulate the phagocytosis process at different levels, which may be necessary to co-ordinate the ability of hemocytes to recognize and engulf their target. Moreover, genes regulated by ecdysone signaling can be implicated in more than one process, for example phagocytosis and AMP expression (e.g. PGRP-LC), or phagocytosis and cell migration (e.g. SCAR); this may contribute to synchronisation of different hemocyte immune functions.

The functional relevance of increased cellular immune activity at metamorphosis is an intriguing question. Recent studies of the contribution of cellular immunity to *Drosophila* defenses have revealed that flies in which hemocytes are genetically ablated present a high lethality at metamorphosis [12], [13]. This is likely the result of opportunistic bacterial infections, as feeding antibiotics was sufficient to restore wild-type viability. We did not observe such lethality under normal conditions when expressing *EcRDN* in hemocytes; however in our hands *Phago^less^* lethality in absence of infection is also lower than that previously described [13]. This suggests that our fly strains and fly food do not harbor the same bacterial types as those used in previous studies, leading to distinct opportunistic infection scenarios. Nevertheless, our data indicate a significant lethality of *HmlΔ>EcRDN* pupae not only after septic injury with *E. faecalis* or *E. carotovora*, but also after oral infection at larval stages with *E. carotovora*, a bacterium that is not usually lethal in wild-type individuals. This lethality is quite dramatic considering only hemocytes express the transgene, and is similar to the lethality in hemocyte-ablated individuals ([12], [13] and this study). It indicates that ecdysone regulation is essential for hemocyte immune functions and survival after infection.

Metamorphosis may represent a stage of predisposition to opportunistic oral infection, as the larval midgut is replaced by the adult intestinal epithelium. We speculate that histolysis of the gut could release bacteria from the lumen into the body cavity; active hemocytes may be required to limit the spreading of bacteria from temporary weak points in the epithelium. We have shown that *HmlΔ>EcRDN* prepupae induce a normal intestinal and systemic humoral immune response after being orally infected at larval stage (Fig. S6 and S7). In the case of both septic injury and oral infection, it is therefore likely that the main cause of decreased survival in *HmlΔ>EcRDN* pupae is their striking hemocyte phagocytosis phenotype, possibly in combination with lack of motility, inability to chemotax to damaged tissue or other potential uncharacterized hemocyte defects.

The synchronization of multiple processes is a fundamental requirement for successful development, and likely to rely on hormonal signaling. Altogether, our data reveal the importance of steroid hormone signaling in the synchronization of development and immunity in *Drosophila*, by ecdysone-dependent activation of hemocytes at pupariation. Rus *et al*. have recently shown [45], that ecdysone signaling affects the humoral response through regulation of PGRP-LC expression. Interestingly, they observed an impact of this regulation on the ability of adult flies to survive infection, indicating that ecdysone regulation of immunity extends beyond metamorphosis. In humans, hormonal activation of macrophages underpins various cancer pathologies [46] and is therefore highly relevant in clinical terms. It is also generally accepted that steroid hormones impact immunity in mammals. For example, glucocorticoids are commonly used in pharmacology for their anti-inflammatory properties [47]. However, their regulation of the immune response is complex, as they can also enhance the immune response [48], [49]. More generally, steroid hormones' specific action on monocytes is still not very well documented, mainly due to the complexity of mammalian systems and experimental limitations. Elucidating mechanisms for steroid hormone regulation of cellular immunity (e.g. [50]) will be essential for a full understanding of sex differences in immunity and inflammation. Here, in a simpler model system, we used an approach that allows *in vivo* and cell autonomous analysis of the hormonal regulation of innate immune cells. We think that in the future, the potential for genetic manipulation in the *Drosophila* model combined with the *in vivo* assays and transcriptomic data developed in this study should aid in deciphering the molecular mechanisms at work behind phagocyte activation by steroids, both in terms of cell migration and immunity *per se*.

## Materials and Methods

### *Drosophila* stocks

We used the following stocks from Bloomington Stock Centre: *w^1118^*; *HmlΔ-GAL4,UAS-GFP* (30142); *UAS-EcRA-DN* (9451, 9452); *UAS-EcRB1-DN* (6869, 6872); *UAS-EcRB2-DN* (9449, 9450); *UAS-EcR-RNAi* (9326, 29374); *USP-RNAi* (27258); and *UAS-Bax/CyO* from [13]. Flies were kept on standard fly food (VDRC recipe). Results obtained with DN^F645A^ are shown in all figures except when specified.

### Imaging

Pupae were mounted and hemocytes imaged live through cuticle as previously described [51]. Images were acquired using a confocal laser line-scanning (LSM 5 Live; Carl Zeiss) or point-scanning (LSM710; Carl Zeiss) microscope (40× oil objective). Mean velocity/min of hemocytes tracked manually over 10 min using the ImageJ plugin ‘Manual Tracker’ was used as a measure of motility. At least 40 cells were tracked per condition, corresponding to at least 4 independent experiments. For tracking plots, trajectories of 20 cells were transformed and analysed using the ImageJ plugin ‘Chemotaxis Tool’ as previously described [49].

### Laser wounding

Wounds were made in prepupae in the dorsal thoracic epithelium using a UV laser controlled by galvanometric mirrors. Laser was emitted at 355 nm as a 20 second train of 1 second square pulses and was fired 9 times to form a square wound of approximately 40×40 µm. The wound was made in a region lateral to the sessile patches, just below the pupal epithelium and always included a tracheal branch. Hemocytes were tracked and hemocytes recruited to the wound over time were counted; the rate of recruitment was calculated as the number of hemocytes recruited to the wound over 150 min after wounding.

### Morphometric analysis by flow cytometry

Hemocytes were bled into PBS and analysed for their FSC-Area and SSC-Area with a CyAn ADP flow cytometry Analyzer (Dako Cytomation/Beckman Coulter). The hemocyte population was defined as GFP positive cells using hemocytes from *w^1118^* as a negative control to establish the threshold.

### Infection, survival and phagocytosis assay

For septic injury, pupae were pricked in the lower abdomen with a needle dipped in bacterial culture. *E.carotovora* (strain *E.c.c.15*) were grown overnight in Luria Bertani (LB) at 29°C and adjusted to O.D. 100. *E. faecalis* were grown overnight in LB at 37°C and adjusted to O.D. 5. Clean pricking with sterilized needle was used as a control. Oral infection of larvae was performed as described ([52] and see SI for more details) by feeding larvae with a mixture of banana and *E.c.c.15*. Pupae were maintained at 29°C and eclosion of adults was scored after 5 days. For Fig. 3A, the stage of death was determined by careful examination and classification between four approximate stages: early pupae (from approx. 12 h to 36 h), intermediate 1 (from approx. 36 h to 54 h), intermediate 2 (from approx. 36 h to 72 h) and late pupal stage (from approx. 72 h to 90 h), corresponding to pupae with the appearance they adopt just before eclosion. For *ex vivo* phagocytosis assay, late L2/young L3 larvae, or white prepupae, were bled into 400 µL PBS (pH 7.4) and 8 µL pHRodo Red *E. coli* BioParticles (1 mg/mL; molecular Probes) were added. Phagocytosis was assayed 30 min later by flow cytometry. For *in vivo* assay, 80 nL of pHRodo particles were injected in prepupae 1h30 APF, using a nanoinjector (Nanoliter 2000, World Precision Instruments). Prepupae were either imaged 1 h after injection, or bled into PBS and analyzed by flow cytometry with a modular Flow Cytometer (MoFlo; Dako Cytomation), using 561 nm and 488 nm lasers. pHRodo particles in solution, *w^1118^* non-injected flies, *HmlΔ>GFP* non-injected flies and *w^1118^* flies injected with pHRodo particles were used to define the thresholds for GFP and phagocytosed red-particle emission. The experiment was repeated three times, using at least 10 prepupae (roughly 10 000 hemocytes) per genotype and experiment. For *in vivo* phagocytosis assay with live bacteria, 50.6 nL of *E. coli-RFP* (at O.D. 2 after dilution in PBS) were injected in prepupae 1h30 APF. After 10 min recovery, prepupae were mounted, and imaged at 20 min post-injection. The central-most sessile patch was imaged at 40× to a depth of 30 µm. Phagocytosis was scored by eye from z-stacks; *E.coli-RFP* were scored as either attached or internalized and phagocytic index was calculated using total hemocyte number.

### Phalloidin and DAPI staining; measure of dead cell phagocytosis

Circulating hemocytes were retrieved by bleeding animals into 20 µL of PBS. After 20 min, they were fixed in a 4% formaldehyde solution for 15 min, then washed in PBST (0.1% Triton X-100 in PBS) and incubated during 30 min at room temperature in a blocking solution (PBST +1% Bovine Serum Albumin). Bleeds were incubated with phalloidin (1∶200 µL; Invitrogen) and DAPI (1 µm/mL) for 30 min, then washed for 1 h in PBS and mounted in mounting medium. Quantification of hemocytes phagocytosing muscle tissue was measured as the percentage of hemocytes positive for phalloidin-positive vacuoles among total hemocyte number.

### Statistical analysis

Statistical significance was defined by pair-wise comparison to controls using the Mann–Whitney U test (non parametric), *t*-test (parametric), or Wilcoxon test (survival experiments). Indicated *p*-values are two-tailed. Calculations and graphs were produced using Excel (Microsoft) and Prism (Graphpad). Symbols in the figures: *p<0.05; **p<0.01; ***p<0.001; ns: not significant.

### FACS-sorting, RNA isolation, target synthesis and hybridization to Affymetrix Array Strips

Hemocytes were isolated by FACS from 3rd instar larvae (at the late feeding stage) and prepupae (1 h to 2 h APF) corresponding to two different genotypes: *w; HmlΔGal4, UAS-GFP/+* (as control), *w; HmlΔGal4; UAS-GFP/UAS-EcRB1DN^W650^*. For each of the four conditions we performed three biological replicates. Hemocytes were sorted based on their fluorescence and directly into the lysis buffer prior to total RNA extraction using the RNeasy Plus Micro Kit (Qiagen). Concentration and purity was determined by spectrophotometry and integrity was confirmed using an Agilent 2100 Bioanalyzer with a RNA 6000 Nano Assay (Agilent Technologies).

RNA was processed for use on Affymetrix (Santa Clara) Drosophila Gene 1.1 ST Array Strips using the Ambion WT Expression Kit (Life Technologies) and Affymetrix GeneChip WT Terminal Labeling Kit, according to the manufacturer's protocols. Briefly, 100 ng of total RNA containing spiked in Poly-A RNA controls (GeneChip Expression GeneChip Eukaryotic Poly-A RNA Control Kit; Affymetrix) was used in a reverse transcription reaction (Ambion WT Expression Kit) to generate first-strand cDNA. After second-strand synthesis, double-stranded cDNA was used in an *in vitro* transcription (IVT) reaction to generate cRNA (Ambion WT Expression Kit). 15 µg of this cRNA was used for a second cycle of first-strand cDNA synthesis (Ambion WT Expression Kit). 5.5 µg of single stranded cDNA was fragmented and end-labeled (GeneChip WT Terminal Labeling Kit; Affymetrix). Size distribution of the fragmented and end-labeled cDNA, respectively, was assessed using an Agilent 2100 Bioanalyzer with a RNA 6000 Nano Assay.

3.5 µg of end-labeled, fragmented cDNA was used in a 150-µl hybridization cocktail containing added hybridization controls (GeneAtlas Hybridization, Wash, and Stain Kit for WT Array Strips, Affymetrix), of which 120 µl were hybridized on array strips for 20 h at 48°C. Standard post hybridization wash and double-stain protocols (GeneAtlas Hybridization, Wash, and Stain Kit for WT Array Strips, Affymetrix) were used on an Affymetrix GeneAtlas system, followed by scanning of the array strips.

### Microarray data analysis

The 12 scanned arrays were analyzed first with Affymetrix Expression Console software using RMA to obtain expression values and for quality control. Control probe sets were removed and log2 expression values of the remaining 15391 transcripts were imported into Chipster 2.4 [53]. Differential expression was determined by empirical Bayes two-group test [54] with Benjamini-Hochberg multiple testing correction and a p-value cut-off of 0.001.

### Quantitative PCR (qPCR)

Hemocytes were sorted and RNA extracted as described for microarrays. RNA concentration was measured with a Nanodrop 1000 spectrophotometer. Complementary DNA was synthesized using Transcriptor First Strand cDNA Synthesis kit (Roche). For quantitative PCR, kit from Applied Biosystems was used (ViiA 7 System, Applied Biosystems). See supplementary information (Text S1) for primer sequences and protocol for qPCR analysis of AMP expression in gut and whole pupa.

## Supporting Information

Figure S1 **Ecdysone-dependent evolution of hemocyte granularity and size over metamorphosis.** A. Forward scatter (FSC)–Area/side scatter (SSC)–Area plots reflecting size (x axis) and granularity (y axis) of hemocytes retrieved from L3W (blue), 8 h APF (orange) and 18 h APF (brown) control and *HmlΔ>EcRB1DN* animals. Note, this is the same data represented in Fig. 1E and F, separated into distinct plots for each stage for clarity. (B–C) FSC-A (indicative of cell size; B) and SSC-A (indicative of cell granularity; C) histograms for populations of control hemocytes (top) and EcRB1DN-expressing hemocytes (bottom) at different time points before (L3W larvae) and after puparium formation. Control hemocytes present a clear shift in FSC-A and SSC-A over development while the EcRB1DN-expressing hemocytes retain a larval size and morphology. These data encompass the data presented in Fig. 1E and F and in Fig. S1A.
(TIF)

Figure S2 **Hemocytes expressing EcRB1 DN proliferate more actively in late 3^rd^ instar larvae.** L3W larvae were bled and proliferation was evaluated by measurement of the percentage of cells positive for a Phospho-histone H3 (PH3) staining among the hemocyte population (t-test; P<0.0001).
(TIF)

Figure S3 **Neither embryonic hemocyte dispersal nor recruitment of larval hemocytes to the gut proventriculus are affected by the expression of EcRB1DN.** (A–B) Similar dispersal of hemocytes in *w; srp^hemo^Gal4, UAS-GFP/+* embryo (A) or *w; srp^hemo^Gal4, UAS-GFP/UAS-EcRB1DN* embryo (B). The *serpent^hemo^Gal4* (*srp^hemo^*) driver was chosen for its early expression in hemocytes. (C–D) Similar numbers of hemocytes are recruited to the proventriculus in *w; HmlΔGal4, UAS-GFP/+* (C) and *w; HmlΔGal4, UAS-GFP/UAS-EcRB1DN* (D) larvae. In all pictures, anterior is up. Scale bars represent 20 µm.
(TIF)

Figure S4 **Expression of** ***EcRB1DN*** **less than 18 h before puparium formation is sufficient to affect hemocyte motility.** We used a temperature sensitive (ts) Gal80, a Gal4 inhibitor, to control the expression of the *EcRB1DN* transgene in time. *HmlΔGal4, UAS-GFP; tub-Gal80^ts^/+* (*Gal80^ ts^* control) and *HmlΔGal4, UAS-GFP; tub-Gal80^ ts^/EcRB1DN* (*Gal80^ ts^ EcRB1DN*) larvae were grown at 18°C (permissive for Gal80_ts_), transferred to 29°C (restrictive for Gal80*^ts^* - *EcRB1DN* is expressed) at late larval stage and hemocyte motility was measured 18 h later in 1 h APF-prepupae. P<0.001. Mean and SEM are displayed.
(TIF)

Figure S5 **Pupae in which hemocytes express EcRB1DN survive metamorphosis and are not delayed in their pupal development.** (A) Lethality at metamorphosis was very low and similar between control individuals and individuals which hemocytes express EcRB1DN (Wilcoxon test; P = 0.4042). Survival over metamorphosis is represented as the percentage of prepupae giving rise to adults. (B) The time needed for metamorphosis was not affected by expression of EcRB1DN in hemocytes (Wilcoxon Test; P = 0.7792). The curve represents the percentage of prepupae eclosed at different time points APF. Experiments were performed at 29°C.
(TIF)

Figure S6 **Expression of EcRB1DN in hemocytes does not affect the humoral systemic immune response to oral infection by** ***E. carotovora*** **.** *HmlΔ>GFP* (control) and *HmlΔ>GFP, EcRB1DN* L3 larvae were fed on banana mixed with LB medium (as a non-infected control) or on banana mixed with a culture of *E.carotovora*. Prepupae at two stages – early (light prepupae, 0–3 h APF) and late (dark prepupae, 4–8 h APF) were assessed for the induction of the humoral systemic immune response by RNA extraction from whole prepupae and quantitative PCR on the AMPs *Diptericin, Attacin, Metchnikowin* and *Drosomycin* (see Text S1 for details). In both genetic contexts, the transcription of all AMPs was strongly induced after infection except for *Drosomycin* (an antifungal AMP); a stronger induction was observed in late (dark) prepupae. Importantly, no significant differences in expression of any of the AMPs tested were observed between control and EcRB1DN, except for Attacin, which expression was significantly higher in *HmlΔ>GFP, EcRB1DN* late prepupae (p<0.05). These data correspond to three independent biological repeats. Mean and SEM are displayed.
(TIF)

Figure S7 **Expression of EcRB1DN in hemocytes does not affect the local epithelial immune response to oral infection by** ***E. Carotovora*** **.** *HmlΔ>GFP* (control) and *HmlΔ>GFP, EcRB1DN* L3 larvae were fed on banana mixed with LB medium (control) or on banana mixed with a culture of *E.carotovora*. Prepupae were assessed for the induction of the local epithelial immune response by RNA extraction from guts dissected at 1–4 h APF, and quantitative PCR on the AMP *Diptericin* (see Text S1). In both genetic contexts, the immune response was strongly induced after infection and *HmlΔ>GFP, EcRB1DN* prepupae induced expression to a similar extent as controls. This graph corresponds to two independent biological repeats. *Dpt* expression is normalized to *Ecc15*-infected control and mean and data range are displayed.
(TIF)

Figure S8 **An hemocyte has internalized an** ***E.coli-RFP*** **bacteria.** This image corresponds to an orthogonal cut of a still from video S5, last time point (t = 31), showing a red *E.coli-RFP* bacteria inside a green hemocyte (GFP).
(TIF)

Table S1 **Genes for which expression is significantly changed at metamorphosis in an EcR-dependent manner.** From the microarray data, we selected genes for which expression was significantly changed (up or down-regulated; p<0.001) at metamorphosis in control hemocytes. We then restricted our list to genes for which expression at pupal stage was significantly different between control and EcRDN-expressing hemocytes (p<0.001).
(XLSX)

Table S2 **Microarray data.** Data obtained from the microarrays, with four conditions (control and EcRDN-expressing hemocytes, at larval and pupal stage) including three biological repetitions each. The table also presents the FC and p values for the following comparisons: control pupa vs control larva, EcRDN pupa vs EcRDN larva, EcRDN larva vs control larva and EcRDN pupa vs control pupa.
(XLSX)

Table S3 **EcR transcriptionally regulates genes involved in cell motility.** Genes for which expression is significantly changed at metamorphosis in an EcR-dependent manner (Table S1) were cross-referenced with genes associated with a GO term related to cell motility (GO terms used: cell migration, cell motility, cell chemotaxis).
(XLSX)

Table S4 **EcR transcriptionally regulates genes involved in cell shape regulation.** Genes for which expression is significantly changed at metamorphosis in an EcR-dependent manner (Table S1) were cross-referenced with the results of screens performed on S2 cells for changes in cell morphology (published in [41], [42]).
(XLSX)

Table S5 **List of primers used for RT-qPCR.**
(DOC)

Text S1 **Supplementary material and methods.** Material and methods used to perform the experiments presented as supplementary figures.
(DOC)

Video S1 **Hemocytes from a control prepupa acquire motility upon metamorphosis.** Hemocytes visible under the dorsal epithelium in a control prepupa (*HmlΔ>GFP*) were imaged for 3 h, from 1 h APF to 4 h APF. The hemocytes can be observed changing morphology, and disperse by migrating away from their original location. Time indicated on the film is time APF. Images were analyzed by time-lapse confocal microscopy using a laser-scanning confocal microscope (LSM510; Carl Zeiss). Frames were taken every minute and are displayed at a rate of 7 frames/second. The scale represents 20 µm.
(MOV)

Video S2 **Hemocytes blocked in ecdysone reception do not acquire motility at metamorphosis.** Hemocytes visible under the dorsal epithelium in an *HmlΔ>GFP, EcRB1DN* prepupa were imaged for 3 h, from 1 h APF to 4 h APF. These hemocytes, which express a dominant negative form of the EcRB1, barely move and do not change morphology. Time indicated on the film is time APF. Images were analyzed by time-lapse confocal microscopy using a laser-scanning confocal microscope (LSM510; Carl Zeiss). Frames were taken every minute and are displayed at a rate of 7 frames/second. The scale represents 20 µm. Stills from this video are presented in figure 2.
(MOV)

Video S3 **Prepupal ‘sessile’ hemocytes are recruited to wound sites.** A wound (indicated by a box) was made with a laser in the vicinity of a sessile patch in a control prepupa (*HmlΔ>GFP*), at 1h30 APF. Hemocytes were imaged for 3h20 after wounding. Time indicated on the film is time APF; the film starts immediately after wounding. The hemocytes respond to the wound by chemotaxing towards it, the majority originating from the lateral and longitudinal sessile patches. Images were analyzed by time-lapse confocal microscopy using a laser-scanning confocal microscope (LSM510; Carl Zeiss). Frames were taken every minute and are displayed at a rate of 14 frames/second. The scale represents 20 µm. Stills from this video are presented in figure 3.
(MOV)

Video S4 **Hemocytes insensitive to ecdysone are impaired in their recruitment to wounds.** A wound (indicated by a box) was made with a laser in the vicinity of a sessile patch in a *HmlΔ>GFP*, *EcRB1DN* prepupa, at 90 min APF. Hemocytes were imaged for 2h20 after wounding, from 1h30 APF to 3h50 APF. Time indicated on the film is time APF; the film starts immediately after wounding. Very few hemocytes can be visualised being recruited to the wound. Images were analyzed by time-lapse confocal microscopy using a laser-scanning confocal microscope (LSM510; Carl Zeiss). Frames were taken every minute and are displayed at a rate of 7 frames/second. The scale represents 20 µm.
(MOV)

Video S5 **Example of** ***in vivo*** **live imaging of phagocytosis.** A hemocyte in a control prepupa was filmed performing attachment, engulfment and internalization of an *E.coli-RFP* bacteria. One can see other hemocytes in the act of phagocytosis, or with already internalized bacteria, as well as free bacteria propelled by the hemolymph circulation. Hemocytes are filmed live through the cuticle of the prepupa. Images were acquired every 15 seconds. The film is displayed at 10 fpm and corresponds to approximately 8 minutes.
(AVI)

Video S6 **3D-image of an hemocyte imaged live in the act of engulfing a bacteria.** This 3D image corresponds to the time point 17 of video S5. The hemocytes is in green (GFP), the bacteria in red (*E.coli-RFP*). 3D projection was realized with the corresponding ImageJ plugin.
(AVI)

Video S7 **Pupal hemocytes are not carried in the hemolymph by the movement of the heart.** A pupa is filmed over several minutes, where heartbeat pulses can be observed. Hemocytes are attached to surrounding tissues and do not move with the pulses.
(MOV)

## References

[1] Kollmann TR, Levy O, Montgomery RR, Goriely S (2012) Innate Immune Function by Toll-like Receptors: Distinct Responses in Newborns and the Elderly. Immunity 37: 771–783. 23159225 10.1016/j.immuni.2012.10.014 PMC3538030

[2] Becker T, Loch G, Beyer M, Zinke I, Aschenbrenner AC, et al (2010) FOXO-dependent regulation of innate immune homeostasis. Nature 463: 369–373. 20090753 10.1038/nature08698

[3] Cohen S, Janicki-Deverts D, Doyle WJ, Miller GE, Frank E, et al (2012) Chronic stress, glucocorticoid receptor resistance, inflammation, and disease risk. Proc Natl Acad Sci U S A 109: 5995–5999. 22474371 10.1073/pnas.1118355109 PMC3341031

[4] Silver AC, Arjona A, Walker WE, Fikrig E (2012) The Circadian Clock Controls Toll-like Receptor 9-Mediated Innate and Adaptive Immunity. Immunity 36: 251–261. 22342842 10.1016/j.immuni.2011.12.017 PMC3315694

[5] Stone EF, Fulton BO, Ayres JS, Pham LN, Ziauddin J, et al. The circadian clock protein timeless regulates phagocytosis of bacteria in Drosophila. PLoS Pathog 8: e1002445. 10.1371/journal.ppat.1002445 PMC3257305 22253593

[6] Fantuzzi G, Faggioni R (2000) Leptin in the regulation of immunity, inflammation, and hematopoiesis. J Leukoc Biol 68: 437–446. 11037963

[7] Gilliver SC (2010) Sex steroids as inflammatory regulators. J Steroid Biochem Mol Biol 120: 105–115. 20045727 10.1016/j.jsbmb.2009.12.015

[8] Lemaitre B, Hoffmann J (2007) The host defense of Drosophila melanogaster. Annu Rev Immunol 25: 697–743. 17201680 10.1146/annurev.immunol.25.022106.141615

[9] Stuart LM, Boulais J, Charriere GM, Hennessy EJ, Brunet S, et al (2007) A systems biology analysis of the Drosophila phagosome. Nature 445: 95–101. 17151602 10.1038/nature05380

[10] Stuart LM, Ezekowitz RA (2008) Phagocytosis and comparative innate immunity: learning on the fly. Nat Rev Immunol 8: 131–141. 18219310 10.1038/nri2240

[11] Avet-Rochex A, Bergeret E, Attree I, Meister M, Fauvarque MO (2005) Suppression of Drosophila cellular immunity by directed expression of the ExoS toxin GAP domain of Pseudomonas aeruginosa. Cell Microbiol 7: 799–810. 15888083 10.1111/j.1462-5822.2005.00512.x

[12] Charroux B, Royet J (2009) Elimination of plasmatocytes by targeted apoptosis reveals their role in multiple aspects of the Drosophila immune response. Proc Natl Acad Sci U S A 106: 9797–9802. 19482944 10.1073/pnas.0903971106 PMC2700997

[13] Defaye A, Evans I, Crozatier M, Wood W, Lemaitre B, et al (2009) Genetic ablation of Drosophila phagocytes reveals their contribution to both development and resistance to bacterial infection. J Innate Immun 1: 322–334. 20375589 10.1159/000210264

[14] Elrod-Erickson M, Mishra S, Schneider D (2000) Interactions between the cellular and humoral immune responses in Drosophila. Curr Biol 10: 781–784. 10898983 10.1016/s0960-9822(00)00569-8

[15] Kocks C, Cho JH, Nehme N, Ulvila J, Pearson AM, et al (2005) Eater, a transmembrane protein mediating phagocytosis of bacterial pathogens in Drosophila. Cell 123: 335–346. 16239149 10.1016/j.cell.2005.08.034

[16] Matova N, Anderson KV (2006) Rel/NF-kappaB double mutants reveal that cellular immunity is central to Drosophila host defense. Proc Natl Acad Sci U S A 103: 16424–16429. 17060622 10.1073/pnas.0605721103 PMC1637598

[17] Nehme NT, Quintin J, Cho JH, Lee J, Lafarge MC, et al (2011) Relative roles of the cellular and humoral responses in the Drosophila host defense against three gram-positive bacterial infections. PLoS One 6: e14743. 21390224 10.1371/journal.pone.0014743 PMC3048390

[18] Pham LN, Dionne MS, Shirasu-Hiza M, Schneider DS (2007) A specific primed immune response in Drosophila is dependent on phagocytes. PLoS Pathog 3: e26. 17352533 10.1371/journal.ppat.0030026 PMC1817657

[19] Shia AK, Glittenberg M, Thompson G, Weber AN, Reichhart JM, et al (2009) Toll-dependent antimicrobial responses in Drosophila larval fat body require Spatzle secreted by haemocytes. J Cell Sci 122: 4505–4515. 19934223 10.1242/jcs.049155 PMC2787462

[20] Ulvila J, Vanha-aho LM, Kleino A, Vaha-Makila M, Vuoksio M, et al (2011) Cofilin regulator 14-3-3zeta is an evolutionarily conserved protein required for phagocytosis and microbial resistance. J Leukoc Biol 89: 649–659. 21208897 10.1189/jlb.0410195

[21] Krzemien J, Crozatier M, Vincent A (2010) Ontogeny of the Drosophila larval hematopoietic organ, hemocyte homeostasis and the dedicated cellular immune response to parasitism. Int J Dev Biol 54: 1117–1125. 20711989 10.1387/ijdb.093053jk

[22] Basset A, Khush RS, Braun A, Gardan L, Boccard F, et al (2000) The phytopathogenic bacteria Erwinia carotovora infects Drosophila and activates an immune response. Proc Natl Acad Sci U S A 97: 3376–3381. 10725405 10.1073/pnas.070357597 PMC16247

[23] Wu SC, Liao CW, Pan RL, Juang JL (2012) Infection-induced intestinal oxidative stress triggers organ-to-organ immunological communication in Drosophila. Cell Host Microbe 11: 410–417. 22520468 10.1016/j.chom.2012.03.004

[24] Babcock DT, Brock AR, Fish GS, Wang Y, Perrin L, et al (2008) Circulating blood cells function as a surveillance system for damaged tissue in Drosophila larvae. Proc Natl Acad Sci U S A 105: 10017–10022. 18632567 10.1073/pnas.0709951105 PMC2474562

[25] Stramer B, Wood W, Galko MJ, Redd MJ, Jacinto A, et al (2005) Live imaging of wound inflammation in Drosophila embryos reveals key roles for small GTPases during in vivo cell migration. J Cell Biol 168: 567–573. 15699212 10.1083/jcb.200405120 PMC2171743

[26] Wood W, Faria C, Jacinto A (2006) Distinct mechanisms regulate hemocyte chemotaxis during development and wound healing in Drosophila melanogaster. J Cell Biol 173: 405–416. 16651377 10.1083/jcb.200508161 PMC2063841

[27] Bunt S, Hooley C, Hu N, Scahill C, Weavers H, et al (2010) Hemocyte-secreted type IV collagen enhances BMP signaling to guide renal tubule morphogenesis in Drosophila. Dev Cell 19: 296–306. 20708591 10.1016/j.devcel.2010.07.019 PMC2941037

[28] Olofsson B, Page DT (2005) Condensation of the central nervous system in embryonic Drosophila is inhibited by blocking hemocyte migration or neural activity. Dev Biol 279: 233–243. 15708571 10.1016/j.ydbio.2004.12.020

[29] DiAngelo JR, Bland ML, Bambina S, Cherry S, Birnbaum MJ (2009) The immune response attenuates growth and nutrient storage in Drosophila by reducing insulin signaling. Proc Natl Acad Sci U S A 106: 20853–20858. 19861550 10.1073/pnas.0906749106 PMC2791644

[30] Karpac J, Younger A, Jasper H (2011) Dynamic coordination of innate immune signaling and insulin signaling regulates systemic responses to localized DNA damage. Dev Cell 20: 841–854. 21664581 10.1016/j.devcel.2011.05.011 PMC3151532

[31] Storelli G, Defaye A, Erkosar B, Hols P, Royet J, et al (2011) Lactobacillus plantarum promotes Drosophila systemic growth by modulating hormonal signals through TOR-dependent nutrient sensing. Cell Metab 14: 403–414. 21907145 10.1016/j.cmet.2011.07.012

[32] Thummel CS (2001) Molecular mechanisms of developmental timing in C. elegans and Drosophila. Dev Cell 1: 453–465. 11703937 10.1016/s1534-5807(01)00060-0

[33] Lanot R, Zachary D, Holder F, Meister M (2001) Postembryonic hematopoiesis in Drosophila. Dev Biol 230: 243–257. 11161576 10.1006/dbio.2000.0123

[34] Sampson CJ, Williams MJ (2012) Real-time analysis of Drosophila post-embryonic haemocyte behaviour. PLoS One 7: e28783. 22242151 10.1371/journal.pone.0028783 PMC3252279

[35] Dimarcq JL, Imler JL, Lanot R, Ezekowitz RA, Hoffmann JA, et al (1997) Treatment of l(2)mbn Drosophila tumorous blood cells with the steroid hormone ecdysone amplifies the inducibility of antimicrobial peptide gene expression. Insect Biochem Mol Biol 27: 877–886. 9474784 10.1016/s0965-1748(97)00072-6

[36] Sinenko SA, Mathey-Prevot B (2004) Increased expression of Drosophila tetraspanin, Tsp68C, suppresses the abnormal proliferation of ytr-deficient and Ras/Raf-activated hemocytes. Oncogene 23: 9120–9128. 15480416 10.1038/sj.onc.1208156

[37] Zaidman-Remy A, Regan JC, Brandao AS, Jacinto A (2012) The Drosophila larva as a tool to study gut-associated macrophages: PI3K regulates a discrete hemocyte population at the proventriculus. Dev Comp Immunol 36: 638–647. 22085781 10.1016/j.dci.2011.10.013

[38] Franc NC, Dimarcq JL, Lagueux M, Hoffmann J, Ezekowitz RA (1996) Croquemort, a novel Drosophila hemocyte/macrophage receptor that recognizes apoptotic cells. Immunity 4: 431–443. 8630729 10.1016/s1074-7613(00)80410-0

[39] Foley E, O'Farrell PH (2003) Nitric oxide contributes to induction of innate immune responses to gram-negative bacteria in Drosophila. Genes Dev 17: 115–125. 12514104 10.1101/gad.1018503 PMC195964

[40] Bai J, Uehara Y, Montell DJ (2000) Regulation of invasive cell behavior by taiman, a Drosophila protein related to AIB1, a steroid receptor coactivator amplified in breast cancer. Cell 103: 1047–1058. 11163181 10.1016/s0092-8674(00)00208-7

[41] Kiger AA, Baum B, Jones S, Jones MR, Coulson A, et al (2003) A functional genomic analysis of cell morphology using RNA interference. J Biol 2: 27. 14527345 10.1186/1475-4924-2-27 PMC333409

[42] Rohn JL, Sims D, Liu T, Fedorova M, Schock F, et al (2011) Comparative RNAi screening identifies a conserved core metazoan actinome by phenotype. J Cell Biol 194: 789–805. 21893601 10.1083/jcb.201103168 PMC3171124

[43] Flatt T, Heyland A, Rus F, Porpiglia E, Sherlock C, et al (2008) Hormonal regulation of the humoral innate immune response in Drosophila melanogaster. J Exp Biol 211: 2712–2724. 18689425 10.1242/jeb.014878 PMC2522372

[44] Zhang Z, Palli SR (2009) Identification of a cis-regulatory element required for 20-hydroxyecdysone enhancement of antimicrobial peptide gene expression in Drosophila melanogaster. Insect Mol Biol 18: 595–605. 19754738 10.1111/j.1365-2583.2009.00901.x

[45] Rus F, Flatt T, Tong M, Aggarwal K, Okuda K, et al (2013) Ecdysone triggered PGRP-LC expression controls Drosophila innate immunity. EMBO J 32: 1626–1638. 23652443 10.1038/emboj.2013.100 PMC3671248

[46] Harkonen PL, Vaananen HK (2006) Monocyte-macrophage system as a target for estrogen and selective estrogen receptor modulators. Ann N Y Acad Sci 1089: 218–227. 17261769 10.1196/annals.1386.045

[47] Necela BM, Cidlowski JA (2004) Mechanisms of glucocorticoid receptor action in noninflammatory and inflammatory cells. Proc Am Thorac Soc 1: 239–246. 16113441 10.1513/pats.200402-005MS

[48] Dhabhar FS (2009) Enhancing versus suppressive effects of stress on immune function: implications for immunoprotection and immunopathology. Neuroimmunomodulation 16: 300–317. 19571591 10.1159/000216188 PMC2790771

[49] Goulding NJ (2004) The molecular complexity of glucocorticoid actions in inflammation - a four-ring circus. Curr Opin Pharmacol 4: 629–636. 15525555 10.1016/j.coph.2004.06.009

[50] Routley CE, Ashcroft GS (2009) Effect of estrogen and progesterone on macrophage activation during wound healing. Wound Repair Regen 17: 42–50. 19152650 10.1111/j.1524-475X.2008.00440.x

[51] Moreira CG, Regan JC, Zaidman-Remy A, Jacinto A, Prag S (2011) Drosophila hemocyte migration: an in vivo assay for directional cell migration. Methods Mol Biol 769: 249–260. 21748681 10.1007/978-1-61779-207-6_17

[52] Romeo Y, Lemaitre B (2008) Drosophila immunity: methods for monitoring the activity of Toll and Imd signaling pathways. Methods Mol Biol 415: 379–394. 18370166 10.1007/978-1-59745-570-1_22

[53] Kallio MA, Tuimala JT, Hupponen T, Klemela P, Gentile M, et al (2011) Chipster: user-friendly analysis software for microarray and other high-throughput data. BMC Genomics 12: 507. 21999641 10.1186/1471-2164-12-507 PMC3215701

[54] Smyth GK (2004) Linear models and empirical bayes methods for assessing differential expression in microarray experiments. Stat Appl Genet Mol Biol 3: Article3. 16646809 10.2202/1544-6115.1027

[55] Philips JA, Rubin EJ, Perrimon N (2005) Drosophila RNAi screen reveals CD36 family member required for mycobacterial infection. Science 309: 1251–1253. 16020694 10.1126/science.1116006

[56] Ramet M, Manfruelli P, Pearson A, Mathey-Prevot B, Ezekowitz RA (2002) Functional genomic analysis of phagocytosis and identification of a Drosophila receptor for E. coli. Nature 416: 644–648. 11912489 10.1038/nature735

[57] Stroschein-Stevenson SL, Foley E, O'Farrell PH, Johnson AD (2006) Identification of Drosophila gene products required for phagocytosis of Candida albicans. PLoS Biol 4: e4. 16336044 10.1371/journal.pbio.0040004 PMC1310651

[58] Kurucz E, Markus R, Zsamboki J, Folkl-Medzihradszky K, Darula Z, et al (2007) Nimrod, a putative phagocytosis receptor with EGF repeats in Drosophila plasmatocytes. Curr Biol 17: 649–654. 17363253 10.1016/j.cub.2007.02.041

[59] Nonaka S, Nagaosa K, Mori T, Shiratsuchi A, Nakanishi Y (2013) Integrin alphaPS3/betanu-mediated phagocytosis of apoptotic cells and bacteria in Drosophila. J Biol Chem 288: 10374–10380. 23426364 10.1074/jbc.M113.451427 PMC3624420

[60] Watson FL, Puttmann-Holgado R, Thomas F, Lamar DL, Hughes M, et al (2005) Extensive diversity of Ig-superfamily proteins in the immune system of insects. Science 309: 1874–1878. 16109846 10.1126/science.1116887

[61] Franc NC, Heitzler P, Ezekowitz RA, White K (1999) Requirement for croquemort in phagocytosis of apoptotic cells in Drosophila. Science 284: 1991–1994. 10373118 10.1126/science.284.5422.1991

[62] Stuart LM, Deng J, Silver JM, Takahashi K, Tseng AA, et al (2005) Response to Staphylococcus aureus requires CD36-mediated phagocytosis triggered by the COOH-terminal cytoplasmic domain. J Cell Biol 170: 477–485. 16061696 10.1083/jcb.200501113 PMC2171464

[63] Rahman M, Haberman A, Tracy C, Ray S, Kramer H (2012) Drosophila mauve mutants reveal a role of LYST homologs late in the maturation of phagosomes and autophagosomes. Traffic 13: 1680–1692. 22934826 10.1111/tra.12005 PMC3528838

[64] Avet-Rochex A, Perrin J, Bergeret E, Fauvarque MO (2007) Rac2 is a major actor of Drosophila resistance to Pseudomonas aeruginosa acting in phagocytic cells. Genes Cells 12: 1193–1204. 17903178 10.1111/j.1365-2443.2007.01121.x

[65] Doherty J, Logan MA, Tasdemir OE, Freeman MR (2009) Ensheathing glia function as phagocytes in the adult Drosophila brain. J Neurosci 29: 4768–4781. 19369546 10.1523/JNEUROSCI.5951-08.2009 PMC2674269

[66] Ziegenfuss JS, Biswas R, Avery MA, Hong K, Sheehan AE, et al (2008) Draper-dependent glial phagocytic activity is mediated by Src and Syk family kinase signalling. Nature 453: 935–939. 18432193 10.1038/nature06901 PMC2493287

