# Supplementary figures and images for: Steroid Hormone Signaling Is Essential to Regulate Innate Immune Cells and Fight Bacterial Infection in Drosophila
Source: PLoS Pathog. 2013 Oct 24;9(10):e1003720. doi: 10.1371/journal.ppat.1003720 (PMC3812043; doi:10.1371/journal.ppat.1003720)

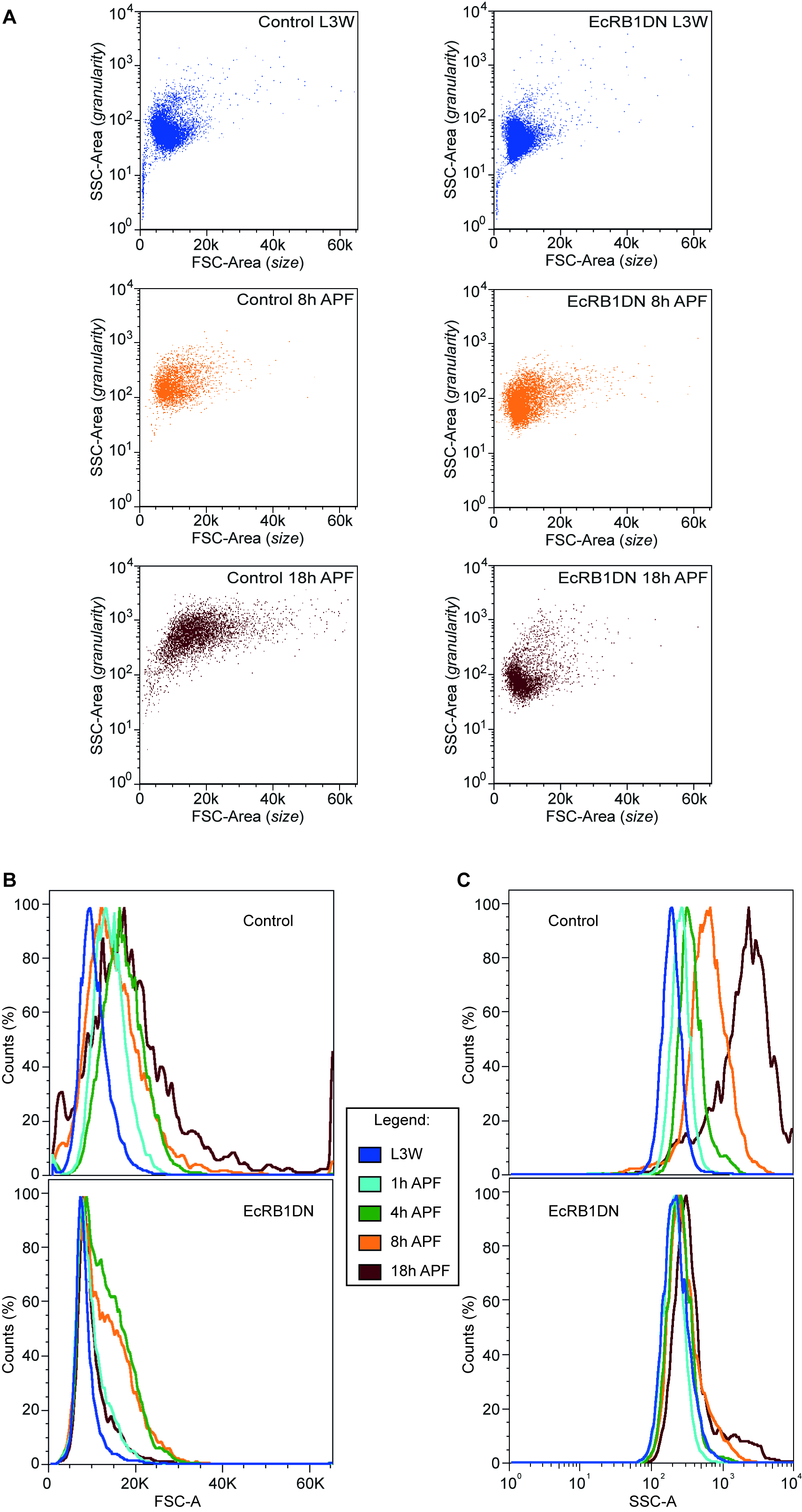

Supplement: Figure S1 — Ecdysone-dependent evolution of hemocyte granularity and size over metamorphosis. A. Forward scatter (FSC)–Area/side scatter (SSC)–Area plots reflecting size (x axis) and granularity (y axis) of hemocytes retrieved from L3W (blue), 8 h APF (orange) and 18 h APF (brown) control and HmlΔ>EcRB1DN animals. Note, this is the same data represented in Fig. 1E and F, separated into distinct plots for each stage for clarity. (B–C) FSC-A (indicative of cell size; B) and SSC-A (indicative of cell granularity; C) histograms for populations of control hemocytes (top) and EcRB1DN-expressing hemocytes (bottom) at different time points before (L3W larvae) and after puparium formation. Control hemocytes present a clear shift in FSC-A and SSC-A over development while the EcRB1DN-expressing hemocytes retain a larval size and morphology. These data encompass the data presented in Fig. 1E and F and in Fig. S1A. (TIF) [file ppat.1003720.s001.tif]

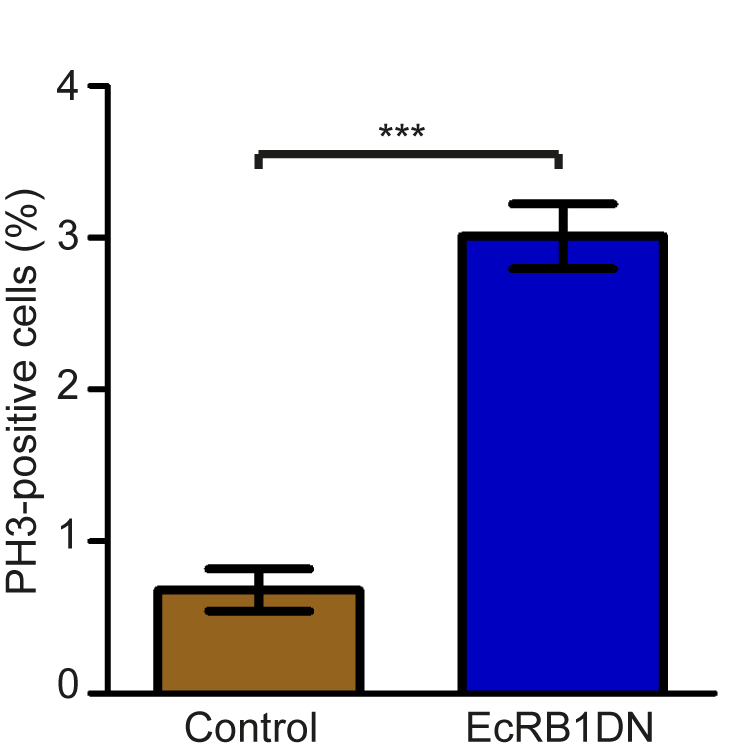

Supplement: Figure S2 — Hemocytes expressing EcRB1 DN proliferate more actively in late 3rd instar larvae. L3W larvae were bled and proliferation was evaluated by measurement of the percentage of cells positive for a Phospho-histone H3 (PH3) staining among the hemocyte population (t-test; P<0.0001). (TIF) [file ppat.1003720.s002.tif]

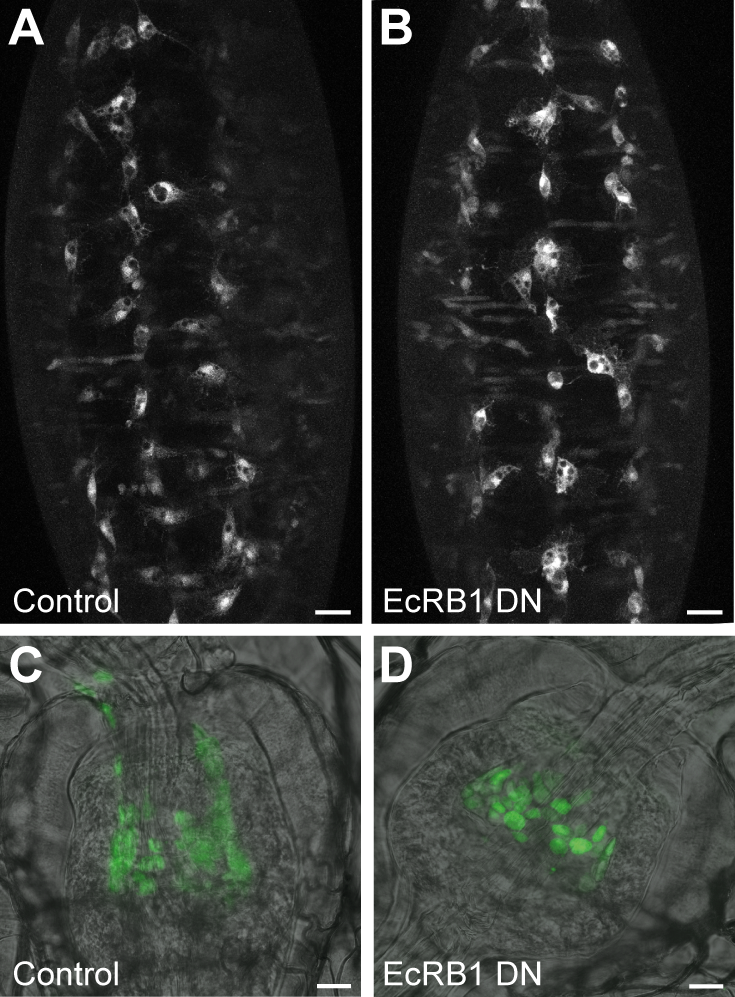

Supplement: Figure S3 — Neither embryonic hemocyte dispersal nor recruitment of larval hemocytes to the gut proventriculus are affected by the expression of EcRB1DN. (A–B) Similar dispersal of hemocytes in w; srphemoGal4, UAS-GFP/+ embryo (A) or w; srphemoGal4, UAS-GFP/UAS-EcRB1DN embryo (B). The serpenthemoGal4 (srphemo) driver was chosen for its early expression in hemocytes. (C–D) Similar numbers of hemocytes are recruited to the proventriculus in w; HmlΔGal4, UAS-GFP/+ (C) and w; HmlΔGal4, UAS-GFP/UAS-EcRB1DN (D) larvae. In all pictures, anterior is up. Scale bars represent 20 µm. (TIF) [file ppat.1003720.s003.tif]

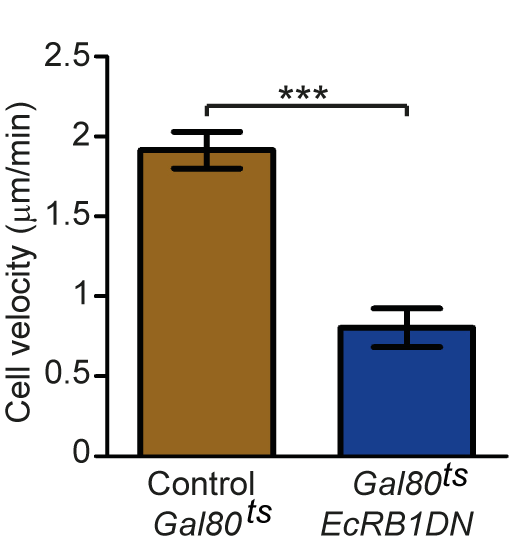

Supplement: Figure S4 — Expression of EcRB1DN less than 18 h before puparium formation is sufficient to affect hemocyte motility. We used a temperature sensitive (ts) Gal80, a Gal4 inhibitor, to control the expression of the EcRB1DN transgene in time. HmlΔGal4, UAS-GFP; tub-Gal80ts/+ (Gal80 ts control) and HmlΔGal4, UAS-GFP; tub-Gal80 ts/EcRB1DN (Gal80 ts EcRB1DN) larvae were grown at 18°C (permissive for Gal80ts), transferred to 29°C (restrictive for Gal80ts - EcRB1DN is expressed) at late larval stage and hemocyte motility was measured 18 h later in 1 h APF-prepupae. P<0.001. Mean and SEM are displayed. (TIF) [file ppat.1003720.s004.tif]

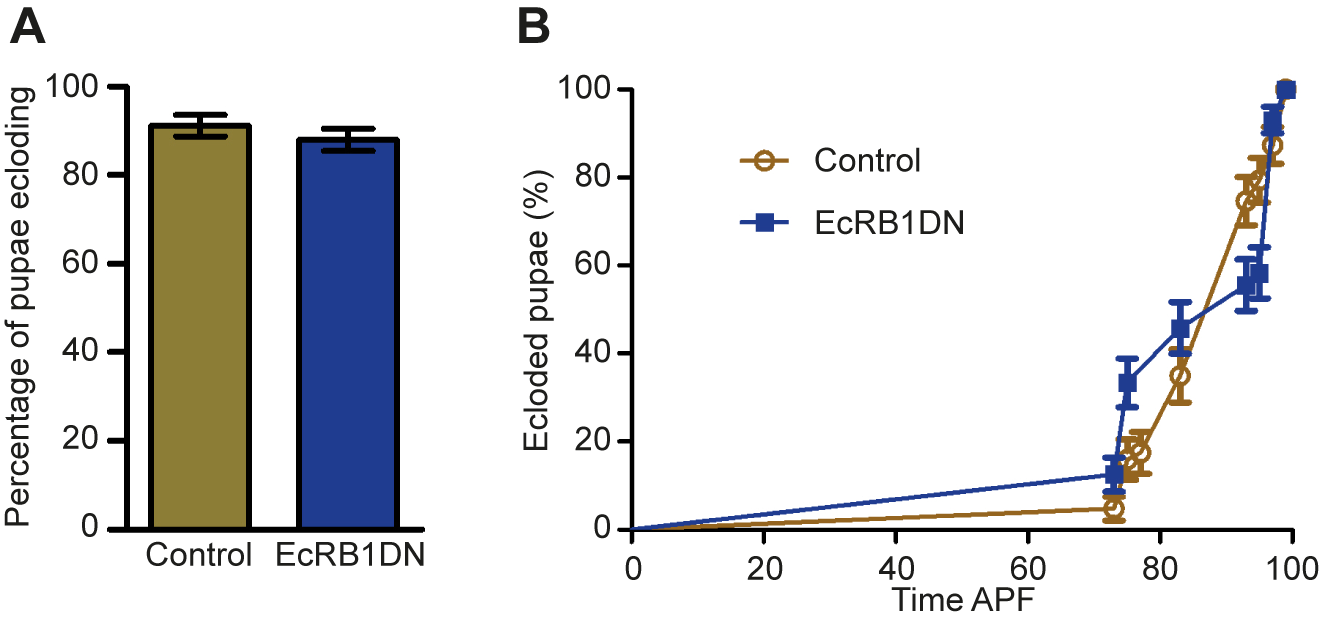

Supplement: Figure S5 — Pupae in which hemocytes express EcRB1DN survive metamorphosis and are not delayed in their pupal development. (A) Lethality at metamorphosis was very low and similar between control individuals and individuals which hemocytes express EcRB1DN (Wilcoxon test; P = 0.4042). Survival over metamorphosis is represented as the percentage of prepupae giving rise to adults. (B) The time needed for metamorphosis was not affected by expression of EcRB1DN in hemocytes (Wilcoxon Test; P = 0.7792). The curve represents the percentage of prepupae eclosed at different time points APF. Experiments were performed at 29°C. (TIF) [file ppat.1003720.s005.tif]

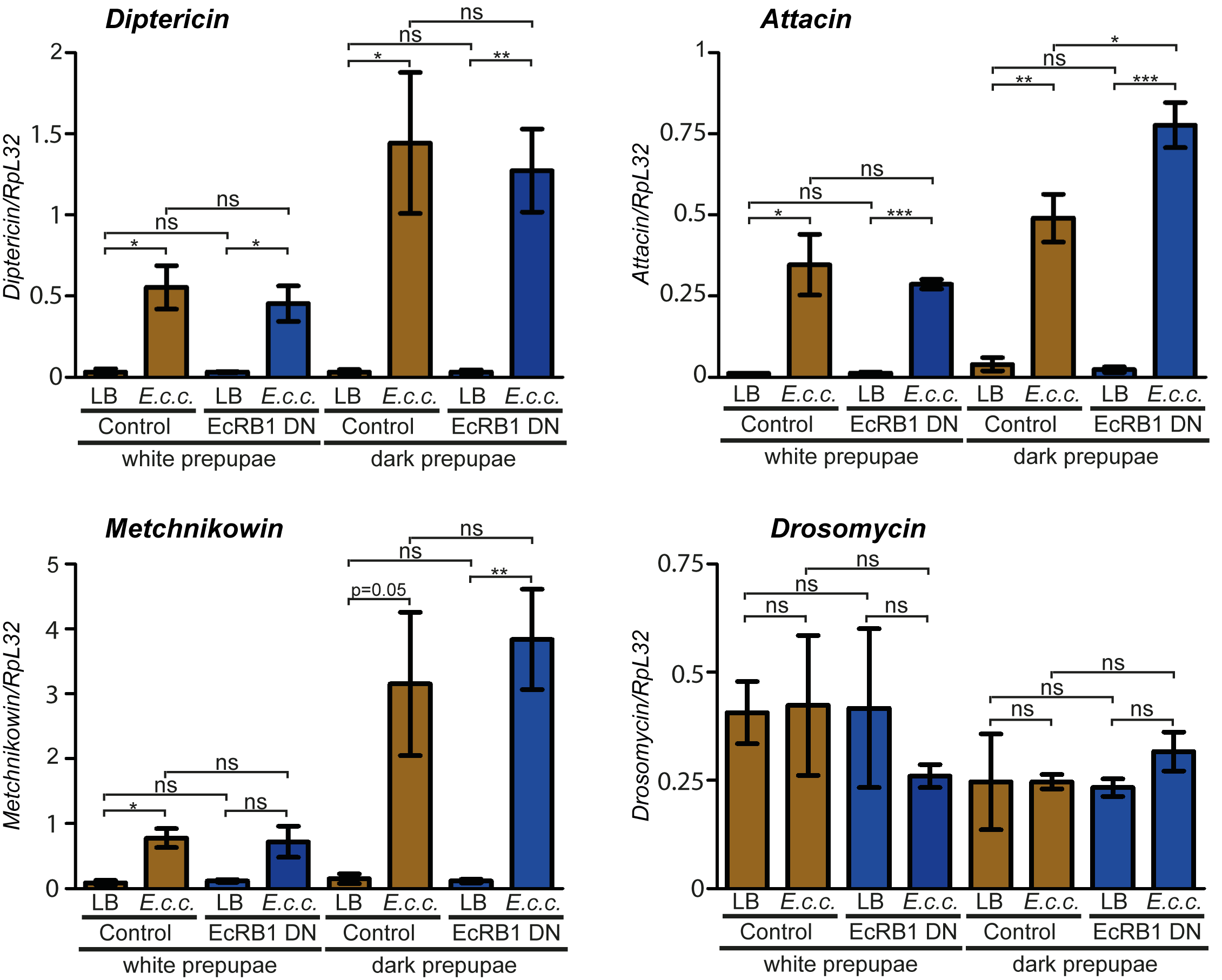

Supplement: Figure S6 — Expression of EcRB1DN in hemocytes does not affect the humoral systemic immune response to oral infection by E. carotovora . HmlΔ>GFP (control) and HmlΔ>GFP, EcRB1DN L3 larvae were fed on banana mixed with LB medium (as a non-infected control) or on banana mixed with a culture of E.carotovora. Prepupae at two stages – early (light prepupae, 0–3 h APF) and late (dark prepupae, 4–8 h APF) were assessed for the induction of the humoral systemic immune response by RNA extraction from whole prepupae and quantitative PCR on the AMPs Diptericin, Attacin, Metchnikowin and Drosomycin (see Text S1 for details). In both genetic contexts, the transcription of all AMPs was strongly induced after infection except for Drosomycin (an antifungal AMP); a stronger induction was observed in late (dark) prepupae. Importantly, no significant differences in expression of any of the AMPs tested were observed between control and EcRB1DN, except for Attacin, which expression was significantly higher in HmlΔ>GFP, EcRB1DN late prepupae (p<0.05). These data correspond to three independent biological repeats. Mean and SEM are displayed. (TIF) [file ppat.1003720.s006.tif]

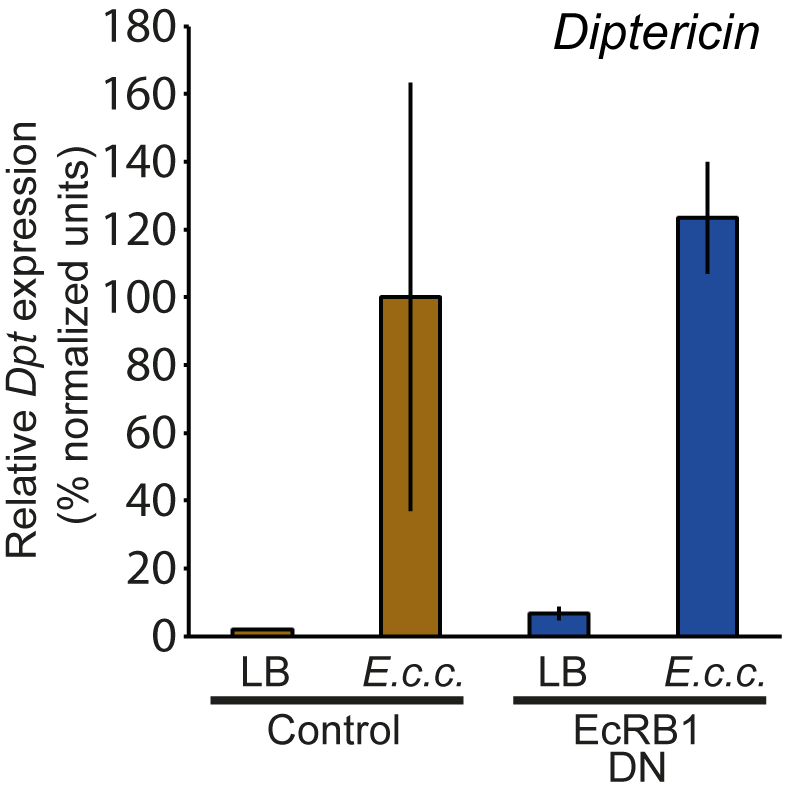

Supplement: Figure S7 — Expression of EcRB1DN in hemocytes does not affect the local epithelial immune response to oral infection by E. Carotovora . HmlΔ>GFP (control) and HmlΔ>GFP, EcRB1DN L3 larvae were fed on banana mixed with LB medium (control) or on banana mixed with a culture of E.carotovora. Prepupae were assessed for the induction of the local epithelial immune response by RNA extraction from guts dissected at 1–4 h APF, and quantitative PCR on the AMP Diptericin (see Text S1). In both genetic contexts, the immune response was strongly induced after infection and HmlΔ>GFP, EcRB1DN prepupae induced expression to a similar extent as controls. This graph corresponds to two independent biological repeats. Dpt expression is normalized to Ecc15-infected control and mean and data range are displayed. (TIF) [file ppat.1003720.s007.tif]

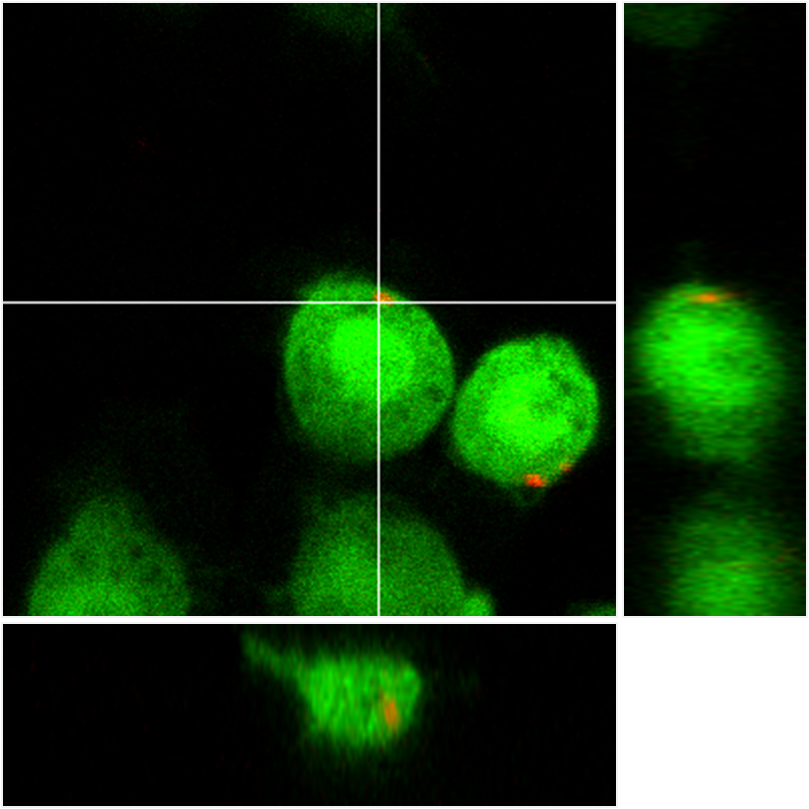

Supplement: Figure S8 — An hemocyte has internalized an E.coli-RFP bacteria. This image corresponds to an orthogonal cut of a still from video S5, last time point (t = 31), showing a red E.coli-RFP bacteria inside a green hemocyte (GFP). (TIF) [file ppat.1003720.s008.tif]
