# Supplementary material for: Steroid Hormone Signaling Is Essential to Regulate Innate Immune Cells and Fight Bacterial Infection in Drosophila
Source: PLoS Pathog. 2013 Oct 24;9(10):e1003720. doi: 10.1371/journal.ppat.1003720 (PMC3812043; doi:10.1371/journal.ppat.1003720)
Supplement: Table S5 — List of primers used for RT-qPCR. (DOC) [file ppat.1003720.s013.doc]

**Table S4. qPCR primers**

|  | **F primer** | **R primer** |
| --- | --- | --- |
| **Diptericin** | GCTGCGCAATCGCTTCTACT | TGGTGGAGTGGGCTTCATG |
| **Attacin** | CCAAGGGCATTGGCAATC | TTTCCGGCGGCGAAA |
| **Metchnikowin** | GCAACTTAATCTTGGAGCGATT | GAAAATGGGTCCCTGGTGA |
| **NimC2** | TGGTTTTACTGAGCGGCCTT | GGCACACACTCGTGAGCTT |
| **Eip93F** | TGCCACGAAAAGTACAGTTGC | TTGCCTGCGACCCATACATT |
| **Eip78C** | GGGTTGCAAGGGATTCTTTCG | GGGAACGCGACCATAACGTA |
| **Tep2** | AGTCGGGCAACGATCTAAGC | GGGTGTCTCGACCCTTCAAA |
| **NimC4** | CGCGAGAAGCAATTCGACAG | GATAGCTTCGTTCAGCCCGA |
| **NimC3** | TGAAGAACCCAGACCGAAGC | CGAGGACCAGCATGGGATAC |
| **Mmp2** | ACCCGAAGGATCTCACTCCA | TGTTGCCAAAAGACTGGAGAC |
| **Dscam** | TTCGTCGAAGTGGTGTCCTG | TGCTAACGGGCTCTGTGATG |
| **crq** | GAGCCCGATGACGACTTCGACATAT | ACCCACTTTTTCGTCACAGTCAGCG |
| **PGRP-LC** | CTGCTGGGTATCGTACTGGC | GGAGCAGACTTCCCTCGATT |
| **CG5697** | CAAGGGGCTACGGCAACTTT | CAGTCCGTAGGAATAGTCAGCA |
| **santa-maria** | CCGAGGAGCCTGTGATTTGT | CCAGAGCCATTTCCTTGTGC |
| **pain** | AGCCAGGTGAACTACATCAACA | GCCGTAGTCCAGCAAGAGTT |
| **GstS1** | GTCGTGACGATCGGTTCAGT | TTGTGCTTCATCGGCCATCT |
| **CG31102** | TGCTAGAAAAGCTGGCGGAA | CCGTGGGATACTGTTCGTGA |
| **eIF-2A** | ATCAGCTCCGAGGATGACGC | GCCGAGACAGACGTTCCAGA |
| **RpL32** | GACGCTTCAAGGGACAGTATCTG | AAACGCGGTTCTGCATGAG |
