# Supplementary material for: Steroid Hormone Signaling Is Essential to Regulate Innate Immune Cells and Fight Bacterial Infection in Drosophila
Source: PLoS Pathog. 2013 Oct 24;9(10):e1003720. doi: 10.1371/journal.ppat.1003720 (PMC3812043; doi:10.1371/journal.ppat.1003720)
Supplement: Text S1 — Supplementary material and methods. Material and methods used to perform the experiments presented as supplementary figures. (DOC) [file ppat.1003720.s014.doc]

**Text S1. Supplementary material and methods**

***Drosophila* stocks**

*srp^hemo^-GAL4 [58]* was obtained from P. Rorth. *HmlΔGal4, UAS-GFP; tub-Gal80ts* was generated using the stock 7018 from Bloomington Center *w*; snaSco/CyO; P{tubP-GAL80ts}.* For Gal80 experiment, *HmlΔGal4, UAS-GFP; tub-Gal80ts/+* and *HmlΔGal4, UAS-GFP; tub-Gal80ts/EcRB1DN* eggs were grown at 18°C (Gal4 activity blocked by Gal80), transferred to 29°C at late larval stage (Gal4 activity released) and hemocyte motility was measured 18h later in 1h APF-prepupae.

**PH3 immunostaining**

After incubation with a blocking solution, samples were incubated overnight at 4°C with primary antibody diluted in blocking solution: rabbit anti-PH3 (1:1000, Sigma); mouse anti-GFP (1:1000, Roche). Samples were washed during 3h, incubated 30min in blocking solution, then 1.5h in secondary antibodies at room temperature: goat anti-rabbit Alexa 568 (1:200, Invitrogen); goat anti-mouse Alexa 488 (1:200, Invitrogen). After 2h of washing, bleeds were incubated in a DAPI solution for 5 min at RT, washed and then mounted.

**Oral infection**

Larvae were transferred to eppendorfs containing banana squash (400 μL) mixed either with 200 μL LB medium (non-infected control) or 200 μL *E. carotovora* (O.D. 200). Eppendorfs were closed with foam plugs for 30 min at room temperature. Larvae were then transferred with their food to standard medium vials and let at 29°C. For survival experiment, upon their pupariation, prepupae were transferred to new vials and survival over metamorphosis assessed.

**Measure of gene expression**

For measure of systemic AMP expression, young prepupae (0-2h APF) or dark prepupae (3-6h APF) were homogenized in 3 ml denaturing solution (4 M guanidine thiocyanate; 25mM sodium citrate pH 7, 0.5% NLauroylsarcosine and, 0.7% β Mercaptoethanol in DEPC-treated water) and RNA extracted using RNeasy Mini kit (Qiagen). RNA concentration was measured with a Nanodrop 1000 spectrophotometer. Complementary DNA was synthesized using Transcriptor First Strand cDNA Synthesis kit (Roche). For quantitative PCR, kit from Applied Biosystems were used with the set of primers described in Table S5 (ViiA 7 System, Applied Biosystems; Roche LightCycler).

For measure of intestinal AMP expression, guts were dissected in ice-cold PBS from 1-4h APF prepupae and pooled in groups of at least 7 guts per biological repeat; 2 biological repeats were performed. Total RNA was extracted in Lysing Matrix D tubes (MP Biomedicals) in a Precellys 24 ribolyser (Bertin Technologies) using Trizol (GIBCO) according to the manufacturers' instructions. RNA concentration was measured with a Nanodrop 1000 spectrophotometer. 1 µg of total RNA was then subjected to DNA digestion using DNAse I (Ambion), immediately followed by reverse transcription using the Superscript II system (Invitrogen) with oligo(dT) primers. Quantitative PCR was performed on a 7900 HT Real-time Quantitative PCR machine (Applied Biosystems), using Fast SYBR Green Master Mix (Life Technologies), following manufacturers' instructions.
